# Supplementary material for: Preventing chronic malnutrition in children under 2 years in rural Angola (MuCCUA trial): protocol for the economic evaluation of a three-arm community cluster randomised controlled trial
Source: BMJ Open. 2023 Dec 18;13(12):e073349. doi: 10.1136/bmjopen-2023-073349 (PMC10748986; doi:10.1136/bmjopen-2023-073349)
Supplement: Author's manuscript [file bmjopen-2023-073349.draft_revisions.pdf]

BMJ Open is committed to open peer review. As part of this commitment we make the peer review history of every article we publish publicly available.

When an article is published we post the peer reviewers' comments and the authors' responses online. We also post the versions of the paper that were used during peer review. These are the versions that the peer review comments apply to.

The versions of the paper that follow are the versions that were submitted during the peer review process. They are not the versions of record or the final published versions. They should not be cited or distributed as the published version of this manuscript.

BMJ Open is an open access journal and the full, final, typeset and author-corrected version of record of the manuscript is available on our site with no access controls, subscription charges or pay-per-view fees (http://bmjopen.bmj.com).

If you have any questions on BMJ Open's open peer review process please email info.bmjopen@bmj.com

BMJ Open

Protocol for the economic evaluation of a three-arm community cluster randomized controlled trial to prevent chronic malnutrition in children under two years in rural Angola (MuCCUA trial)

Journal:	*BMJ Open*
Manuscript ID	bmjopen-2023-073349
Article Type:	Protocol
Date Submitted by the Author:	02-Mar-2023
Complete List of Authors:	Martin-Cañavate, Rocio; Instituto de Salud Carlos III, Centro Nacional de Medicina Tropical<br>Trigo, Elena; Vall d'Hebron University Hospital, Infectious Diseases Department<br>Romay-Barja, María; Instituto de Salud Carlos III, Centro Nacional de Medicina Tropical; Centro de Investigación Biomédica en Red Enfermedades Infecciosas<br>Herrador, Zaida; Instituto de Salud Carlos III (ISCIII), Centro Nacional de Medicina Tropical<br>Aguado, Isabel; Vall d'Hebron University Hospital, Infectious Diseases Department<br>Ramirez, Ferran; Vall d'Hebron University Hospital, Infectious Diseases Department<br>Faria, Lourdes Maria; Fundo Apoio Social-Local Development Institute<br>Silva-Gerardo, Ana; Faculdade de Medicina da Universidade Mandume Ya Ndemufayo<br>Lima, Jose Carlos; Faculdade de Medicina da Universidade Mandume Ya Ndemufayo<br>Iráizoz, Eva; Action Against Hunger Spain<br>Marques, Tayna; Vall d'Hebron University Hospital, Infectious Diseases Department<br>Vargas, Antonio; Action Against Hunger Spain<br>Gomez, Amador; Action Against Hunger Spain<br>Puett, Chloe; Stony Brook University Program in Public Health<br>Molina, Israel; Vall d'Hebron University Hospital, Infectious Diseases Department; Centro de Investigación Biomédica en Red Enfermedades Infecciosas<br>Custodio Cerezales, Estefania; Instituto de Salud Carlos III; Centro de Investigación Biomédica en Red Enfermedades Infecciosas
Keywords:	HEALTH ECONOMICS, Nutrition < TROPICAL MEDICINE, NUTRITION & DIETETICS

For peer review only - http://bmjopen.bmj.com/site/about/guidelines.xhtml

Protocol for the economic evaluation of a three-arm community cluster randomized controlled trial to prevent chronic malnutrition in children under two years in rural Angola (MuCCUA trial)

Rocio Martin-Cañavate[1], Elena Trigo[2], Maria Romay-Barja[1,3], Zaida Herrador[4], Isabel Aguado[2], Ferran Ramirez[2], Lourdes Maria Faria[5], Ana Silva-Gerardo[6], Jose Carlos Lima[6], Eva Iráizoz[7], Tayna Marques[2], Antonio Vargas[7], Amador Gomez[7], Chloe Puett[8], Israel Molina[2,3], Estefania Custodio[1,3*]

Correspondence to Estefanía Custodio ecustodio@isciii.es, +34 622859128, Centro Nacional de Medicina Tropical, Instituto de Salud Carlos III, Avda. Monforte de Lemos, 3 Madrid 28029, Spain

Rocio Martin-Cañavate rocio.mcanavate@isciii.es

Elena Trigo elena.trigo@vhir.org

Maria Romay-Barja mromay@isciii.es

Zaida Herrador zherrador@isciii.es

Isabel Aguado Lobo isabel.aguado@vhir.org

Ferran Ramírez ferran.ramirez@vhir.org

Lourdes Maria Faria Lourdesfaria2003@yahoo.com.br

Ana Silva-Gerardo anasgerardo@yahoo.com.br

Jose Carlos Lima rahita5y@yahoo.com.br

Eva Iráizoz Domínguez eiraizoz@accioncontraelhambre.org

Tayná Marques tmarques@dndi.org

Antonio Vargas avargas@accioncontraelhambre.org

Amador Gómez agomez@accioncontraelhambre.org

Chloe Puett chloepuett@gmail.com

Israel Molina israel.molina@vallhebron.cat

Estefania Custodio ecustodio@isciii.es

Abstract

Introduction: Chronic malnutrition affects 149.2 million children under 5 years of age. It is a serious problem in southern Angola with a prevalence of 49.9% and 37.2% respectively in the provinces of Huila and Cunene. The Crescer project implements a

For peer review only

community-based randomized controlled trial (c-RCT) with three intervention arms, combining standard of care, nutritional supplementation and cash transfers to evaluate their effectiveness in preventing stunting in southern Angola. This protocol describes the planned economic evaluation associated with the c-RCT.

Methods and analysis: A cost-efficiency and cost-effectiveness analysis nested within the MuCCUA trial will be conducted from a societal perspective, measuring programme, provider, institution, participant and household costs. Programme costs will be collected prospectively using a combined calculation method including quantitative and qualitative data. Financial costs will be estimated by applying activity-based costing methods to accounting records using time allocation sheets developed with a standardised tool. Ingredients approach will be used to estimate costs not included in accounting records and indirect costs incurred by beneficiaries through interviews, household surveys and focus group discussions. Cost-efficiency will be estimated as cost per output achieved by combining activity-specific cost data with routine data on program outputs. Cost-effectiveness will be assessed as cost per stunting case prevented. Incremental cost-effectiveness ratios (ICER) will be calculated, comparing the additional cost per improved outcome of the different intervention arms and the standard of care. Sensitivity analyses will be performed to assess robustness of results.

Ethics and dissemination: This economic evaluation will provide useful information to the Angolan Government and other policymakers on the most cost-effective intervention to prevent stunting in this and other comparable contexts. The protocol was approved by the República de Angola Ministério da Saúde Comité de Ética (27C.E/MINSA.INIS/2022) and the findings of this study will be disseminated within academia and the wider policy sphere.

Trial registration number: NCT05571280; pre-results

Keywords: Cost-effectiveness, Stunting, Angola, Chronic malnutrition, multi sectoral nutrition programme, cash transfers, nutritional supplementation

Article Summary

For peer review only - http://bmjopen.bmj.com/site/about/guidelines.xhtml

Strengths and limitations of this study

- This protocol reports planned data collection and analyses of a complex multisectoral community health trial to prevent chronic malnutrition.
- This protocol will contribute to the growing evidence on economic evaluations of multisectoral intervention trials on the prevention of chronic malnutrition.
- The study design draws on a broad and comprehensive cost analysis based on diverse data sources such as trial data, community-level surveys, staff interviews and detailed program expenditures and will allow societal, household and provider costs to be disaggregated.
- The cost estimation of the different arms of the trial will complement the effectiveness results to provide useful information to the Government of Angola and other policymakers on the most cost-effective and cost-efficient intervention to prevent stunting.
- The study is powered to test the cost-effectiveness of each intervention against the control arm only. The study is not powered to assess differences between the interventions as this would require a larger sample size and resources. However, the possibility of a direct comparison between the interventions will be explored.

BACKGROUND

Burden of child undernutrition

Stunting or chronic malnutrition is the impairment of growth and development that children experience due to malnutrition, repeated infections, and inadequate psychosocial stimulation (1). Stunting in early life, particularly in the first 1000 days, from conception through the first two years of life, has adverse physical and functional consequences in the child including higher risk of morbidity and mortality, lower educational attainment and cognition, lower adult earnings, loss of productivity, and, when accompanied by excessive weight gain in childhood, increased risk of nutrition-related chronic diseases in adulthood (2). Stunting affects 149.2 million children under

For peer review only

5 years of age. The number of children with stunting is declining in all regions except Africa (3).

In Angola, the level of chronic malnutrition is high, with 38% of children being stunted; and 5% of children being acutely malnourished nationally (DHS 2016-2017)(3). According to UNICEF, 3.8 million people in Angola face food and nutrition insecurity due to climate shocks (4). Recurrent drought, reduced agricultural and livestock production, crop failures, high food prices and the proliferation of infectious diseases all contribute to compromising maternal and infant nutrition and health (5) (6). The South-Western provinces of Angola have been most severely affected. In the provinces of Cunene and Huila, the latest Integrated Food Security Phase Classification (IPC) analysis revealed that over 1.3 million people are estimated to be experiencing high levels of acute food insecurity with more than 50% of their total population in *Crisis* or *Emergency* levels between April 2021 and March 2022 (6). The situation has deteriorated and will likely worsen during the lean season as food reserves are depleted, drought conditions continue to be exacerbated by erratic below-average rainfall and the humanitarian response proves inadequate to address the most urgent needs (7). Further, a Standardised Monitoring and Assessment of Relief and Transitions (SMART) survey conducted in 2019 in selected municipalities of Huila and Cunene reported a prevalence of global acute malnutrition of 11% in both provinces, and a stunting prevalence of 50% and 37% in the provinces of Huila and Cunene respectively (8).

Due to the severity of the situation, there is a need to improve the nutritional status of this population. In relation to stunting, there is growing evidence that nutrition specific interventions such as small-quantity lipid-based nutrient supplementation (SQ-LNS) have benefits on child growth (9). There is also evidence that nutrition-sensitive interventions, e.g., cash transfers, could improve nutrition and health outcomes and accelerate reductions in stunting (10). The Crescer project through the MuCCUA trial ("Mother and Child Chronic Undernutrition in Angola") is one of the initiatives that aims to address stunting in Southern Angola by implementing a multisectoral approach with both a nutrition-specific and a nutrition-sensitive intervention.

MuCCUA trial

For peer review only - http://bmjopen.bmj.com/site/about/guidelines.xhtml

For peer review only

The Crescer project is an operational research program on the prevention of chronic malnutrition in the southern Angolan provinces of Huila and Cunene. It is embedded in the FRESAN (Fortalecimento da Resiliência e da Segurança Alimentar e Nutricional em Angola) program, funded by the European Union to strengthen resilience and food and nutrition security in South Angola. The Crescer project implements the MuCCUA trial, a three-arm community-based cluster randomized controlled trial of non-inferiority which aims to evaluate the effectiveness and cost-effectiveness of two interventions in preventing stunting in children at 24 months in Huila and Cunene. The comparator arm is defined as the "standard of care". Pregnant women and their newborn children are target recipients of either arm: the comparator arm receives standard of care only, one of the intervention arms receives standard of care plus nutrition supplementation with SQ-LNS and family food ration, and the other intervention arm receives standard of care plus unconditional cash transfers. Community and Health Development Agents (ADECOS) implement the interventions following Angolan government protocols. The primary outcomes are prevalence of stunting and mortality in children. In addition, all study areas (both intervention and control) clusters receive the standard of care. The MuCCUA trial is described in detail elsewhere (11). The purpose of this protocol paper is to fully describe the methodology for the economic evaluation of the trial.

Economic evaluations of multi-sectoral interventions to prevent child stunting

Delivering multi-sectoral interventions combining nutrition-specific and nutrition-sensitive interventions during the first 1000 days of life is key to prevent chronic malnutrition and to improve early child growth (12). Reduction of stunting requires improvements in food and nutrition security, education, WASH (water, sanitation and hygiene interventions), health, the status of women and poverty reduction (2). Maternal and child interventions such as nutritional supplementation with small quantity lipid-based nutrient supplements (13–15), cash transfers (10,16), vitamin A supplementation and behaviour change communication have shown promising results in preventing chronic malnutrition (17). However, evidence on the effectiveness of combining them in multisectoral programs and the costs and cost-effectiveness of nutrition programs incorporating both nutrition-specific and nutrition-sensitive interventions is scarce (18). Though the body of economic evidence supporting nutrition programs is growing, there

For peer review only - http://bmjopen.bmj.com/site/about/guidelines.xhtml

is little evidence of the costs, cost-effectiveness and benefits, and there is no evidence of the additive costs and benefits of the integration of activities across multiple sectors (12). This scarcity of cost-effectiveness data for these interventions is especially acute in low-and middle-income countries (LMICs) (18,19). The diverse methodological challenges that exist across studies and the lack of a standardized method for measuring effectiveness across diverse sectors and outcomes makes it difficult to compare economic evaluations of multisectoral programs.

In addition, multi-sectoral programs including health, nutrition, cash, and behaviour change communication interventions pose challenges for economic evaluations due to their heterogeneity. First, studies with interventions delivered in different sectors can be difficult to compare due to differences in the outcomes and costs included (20–23). Second, the perspective chosen for the economic evaluation poses another challenge for interpretation and comparison of findings between studies because of the different sets of costs included (18,24,25). And third, economic evaluation objectives differ greatly between studies as some aim to prioritize policies and funds, and different studies may aim to inform different decision makers (21). The proposed economic evaluation of the MuCCUA trial aims to address the challenges mentioned above by assessing the cost-effectiveness of the interventions from a societal perspective, including all costs of the interventions regardless of who incurs them.

Aim and objectives

The aim of the MuCCUA trial economic evaluation is to use a societal perspective to estimate the costs, and evaluate the cost-efficiency and cost-effectiveness of three interventions (standard of care, standard of care plus nutritional supplements, and standard of care plus cash transfers) in reducing stunting in children under 2 years of age from poor and very poor households in the southern provinces of Cunene and Huila in Angola.

The specific objectives of the economic evaluation are:

1. To estimate the costs of setting up and implementing each of the MuCCUA interventions.

For peer review only - http://bmjopen.bmj.com/site/about/guidelines.xhtml

2. To calculate costs incurred by the intervention participants and their families as a result of the interventions.
3. To estimate the cost-efficiency of each of the three interventions (e.g., cost per beneficiary contacted, cost per community event conducted, etc.).
4. To estimate the cost-effectiveness as cost per case of stunting averted by comparing incremental costs and incremental changes in the outcomes of the interventions, compared with the standard of care.

In addition to the above objectives, the equity impact analysis of the MuCCUA interventions will be assessed to evaluate how the costs and consequences of the interventions are distributed among different sub-groups of the study population.

METHODS

Study design

A cost-efficiency and cost-effectiveness analysis of the MuCCUA trial, a community-based cluster-randomised trial that compares three interventions to prevent children's stunting taking place between October 2022 and February 2025. We will estimate the total and incremental costs of the interventions prospectively from a societal perspective, measuring programme, provider and household costs.

Patient and Public involvement

Community leaders (Sobas) and the community were involved at the early stages of the research with discussions about the outcomes and the interventions. Then ADECOS were selected by their own communities. Sobas and ADECOS were involved in the cluster randomization. Pregnant women and their families were not involved in setting the research question or the outcome measures, but they were they intimately involved in implementation of the intervention. Methods of recruitment were informed by discussions with Community Activity Assistants and ADECOS through a focus group session. All women from selected randomized communities included were registered and informed of the study by ADECOS.

Participants and their families will be central to dissemination of the baseline information, which will help to motivate community involvement during and beyond the

For peer review only - http://bmjopen.bmj.com/site/about/guidelines.xhtml

study. Once the trial has been published, participants and their communities will be informed of the results through community dialogue.

Study setting and population

The MuCCUA trial is set in four communes of Huila and Cunene, two provinces of South Angola. These provinces are predominantly rural with a combined total population of around 4.4 million (Huila population of 3.185.244 and Cunene population of 1.271.638) (26). There are province-specific differences related to livelihoods. Huila has a higher agricultural productivity and lower food insecurity risk as compared to Cunene where the semi-arid conditions and frequent droughts occasionally result in significant livestock loss, a key livelihood asset. Overall, the population is highly dependent on staple foods (primarily maize, millet and sorghum) and there is poor access to safe drinking water, hygiene and sanitation which may differ by commune also, due to the communes' different geographical features. The socioeconomic status of clusters and households is expected to be homogeneous, as one of the inclusion criteria was to belong to very low levels of poverty. Different ethnic groups are present in the clusters but we do not expect diverse access to interventions according to ethnic origin. However, access to health care and other essential services differs in relation to the distance from the communities to the main settlements of each of the communes. There are remote communities where long distance, lack of transport and lack of trained health professionals and medicines, makes access to basic services inadequate.

The study participants are pregnant women confirmed by pregnancy test, over 16 years of age, and their new-born children. The target population of the interventions is the household in which the pregnant woman and their children live. In the case of having more than one pregnant woman within the household, all women who accept to participate will be included. We consider households to be the person or group of persons, with or without kinship relationships, who have been habitually living under the same roof for at least 6 months or less but with the intention of staying in the residence for the next 6 months and share food and/or other vital needs. Informed cluster-level consent was sought from village leaders to participate in the trial before

For peer review only - http://bmjopen.bmj.com/site/about/guidelines.xhtml

randomization. After acceptance to participate in the study through the informed consent, mothers and their children are followed up for a period of 24 months.

Trial design

In the MuCCUA trial, a total of 36 purposively-selected clusters (the selection of which is described below), with an estimated population of around 55000, were randomized to the three trial arms with 6 clusters per arm in Huíla province, and 6 clusters per arm in Cunene province. Two municipalities (admin 2) were selected per province (admin1) with one commune (admin 3) per municipality (total 2 communes per province). In the province of Huíla, the commune of Libongue (municipality of Chicomba) and the commune of Jamba Sede (municipality of Jamba) were selected. In the province of Cunene, the commune of Otchinjau (municipality of Cahama) and the commune of Mupa (municipality of Cuvelai) were selected. The cluster unit is typically a village or neighbourhood and any nearby hamlets within each commune or, in the case of large neighbourhoods, a distinct part of it that is similar in population size to the unit. The randomisation, which took place in September 2022, was stratified by cluster.

The inclusion criteria for clusters was being considered as one of the municipalities prioritized by the FRESAN program and having a multidimensional poverty level 4 or 5 according to the *Instituto Nacional de Estatísticas* classification (27), not currently having or planned to have in operation other interventions offering monetary or nutritional transfers, acceptance by the municipal and traditional authorities, accessible in a 4x4 vehicle and having a reference health post with reference health personnel.

Recruitment and baseline survey started in October 2022 and will continue until March 2023. Implementation started at recruitment and is planned to end in February 2025. A total of 1440 pregnant women and their newborns, 480 in each arm are enrolled. Primary efficacy analyses will be performed when participant’s children have completed a total of 24 months of follow-up. A full description of the intervention design within the MuCCUA trial is described in detail elsewhere (11). A brief description of the interventions is presented in the following sections.

MuCCUA interventions

Standard of care arm (SOC)

For peer review only - http://bmjopen.bmj.com/site/about/guidelines.xhtml

This is the comparator arm, and communities allocated to this arm only receive the standard of care as described below.

The Ministry of Territorial Development of Angola, through the Institute of Local Development- Social Support Fund (FAS), has created a new national community development policy implementing two new figures: ADECOS and sector supervisors of the main extension technical services (health, social protection, and agriculture). The policy aims to address human resource shortages in remote and rural areas effectively, providing primary and preventive care, improving health status and quality of life in rural communities. This intervention will be used as a comparator as it fulfils the criteria of acceptability, feasibility, relevance and uniformity required to be a good comparator in this type of studies (28). The standard of care activities can be grouped into two blocks.

1) Health promotion activities: including community interventions to promote adequate infant and young child feeding (IYCF) practices, identification and management of malnutrition cases and promotion of adequate hygiene, water and sanitation practices through sensitization activities.

2) Preventive pharmacological activities: including semiannual vitamin A supplementation for children 6-24 months old, semiannual deworming with albendazole for children from 12 months to 24 months of age and pregnant women from the 2nd trimester; and malaria prophylaxis with sulfadoxine-pyrimethamine for pregnant women from 13th week of pregnancy.

The standard of care is implemented in all the communities participating in the study, as detailed below.

Standard of care + Nutritional supplementation (SOC + NUT)

Communities allocated to this arm will receive the standard of care intervention plus nutritional supplementation to families with at least one pregnant woman. Nutritional supplementation will consist of SQ-LNS, a peanut-based ready-to-use home-fortification product to improve diet quality, for the pregnant women and their newborn children, and a complementary food ration for their families. The rations will be as follows: an individual ration of SQ-LNS for pregnant women Enov'Mum (1 sachet of 20 g daily) and Enov'Nutributter for children over 6 months (1 sachet of 20 g daily) produced

For peer review only - http://bmjopen.bmj.com/site/about/guidelines.xhtml

and packaged in France by Nutriset®. A complementary family food ration will comprise of a basket of locally produced staple foods that complements the usual diet (300 Kcal/person/day). The caloric distribution of the basket will be 45% of cereals (corn meal - carbohydrate), 30% of legumes (beans - vegetable protein) and 25% of oil (soybean oil - fat). In addition, 1 kg of iodized salt will be provided.

Standard of care + Cash transfer (SOC + CT)

Communities allocated to this arm will receive the standard of care intervention plus an unconditional cash transfer to families with at least one pregnant woman. A total of 13855 kwanzas/month will be delivered to households with 4 or more inhabitants. A total of 10855 kwanzas/month will be delivered to households with 3 or fewer inhabitants. The amount will be delivered in cash with unconditional format. The amounts of cash were selected based on the amount disbursed by the *Kwenda* project, an Angolan government program funded by the World Bank aiming to fight poverty and to promote sustainable development in the communities with very poor households.

Measurement of health outcomes/effectiveness

The outcomes of the interventions will be assessed through repeated cross-sectional household surveys at baseline, at 6 months of pregnancy, 9 months of pregnancy, 3, 6, 12, 18 months of the newborn child, and end-line at 24 months of newborn child.

The MuCCUA trial will test the effect of each of the experimental interventions (SOC + NUT, SOC + CT arms) relative to the control (SOC arm). A detailed description of the trial outcomes is included in the trial protocol (11).

Primary outcome

The MuCCUA trial primary outcome is prevalence of stunting as measured by height-for-age z-score (HAZ) or length-for-height (LAZ) < − 2 standard deviations (SD) and mean HAZ or LAZ score in children at 24 months of age in the provinces of Huila and Cunene.

The trial sample size was powered to detect a 13% reduction in stunting prevalence. This sample size is sufficient to measure a detectable difference of 10 % between the intervention groups and the comparison group post intervention. Therefore we will conduct a cost-effectiveness analysis for cases of stunting averted if there is evidence of

For peer review only - http://bmjopen.bmj.com/site/about/guidelines.xhtml

impact on the prevalence of stunting in children at 24 months at the 0.1 significance level or below. Cases averted by the two interventions relative to the control group for the outcome will be calculated at 24 months.

Secondary outcomes

The MuCCUA trial has a number of secondary outcomes including: infant mortality rate at 3, 6 , 12, 18 and 24 months, proportion of neonatal low birth weight, and low birth weight for gestational age, proportion of children with anaemia, cumulative incidence of morbidity (malaria, diarrhoea and pneumonia), primary caregiver's knowledge, attitudes and practices related to perinatal and children's caring practices including breastfeeding and hygiene and sanitation, and women and children minimum dietary diversity.

Equity impact

In addition to calculating the efficiency and cost-effectiveness of the MuCCUA trial, an equity impact of the interventions will be conducted to assess whether costs and consequences of the interventions are equitably shared among the target population. The economic evaluation will be stratified through subgroup analyses of the primary and secondary outcomes based on differences of the study population characteristics mentioned earlier. Disaggregation of the findings will be performed in four subgroups per treatment arm, based on province and commune context. In each of these subgroups community group discussions will be carried out with different samples of participants according to their access to basic services and distance to the distribution points of the interventions.

Identification, measurement and valuation of resource use

The cost-efficiency and cost-effectiveness of the MuCCUA interventions will be measured from a societal perspective (24,25); taking into account costs incurred by the implementing agencies, namely the Institute of Local Development-Social Support Fund of Angola (FAS) and Vall d'Hebron Institute of Research (VHIR) (programme-provider costs), ADECOS (community frontline workers) and by the users who are intervention participants and their households. Resource use will be measured using financial expenditure data combined with micro-costing to allow for estimation of both financial

For peer review only - http://bmjopen.bmj.com/site/about/guidelines.xhtml

and economic costs. All cost data will be collected prospectively from the launch of the MuCCUA community trial project for a 3-year (2022-2025) time period, and divided into start-up and recurrent costs. Table 1 provides an overview of the resource use and cost measures that are described in more detail further below.

For peer review only

For peer review only - http://bmjopen.bmj.com/site/about/guidelines.xhtml

Table 1 Overview of resource use and cost measures included in the economic evaluation of the MuCCUA interventions

**Perspective/cost category**	**Type of costs**	**Description**	**Source**	**Sample size**
Programme/ implementing agency	Direct	Costs of implementing the intervention	1. Project accounts of the implementing agencies<br>2. Interviews with the project staff	N/A
	Indirect	Opportunity cost of donated items, volunteer time	1. Interviews with the project staff<br>2. Project records on volunteer involvement<br>3. Project accounts	N/A
Provider/ health system o (Health providers)	Direct	Changes in demand/ utilization of nutrition and health services / Costs of referrals	1. Baseline and endline cross-sectional surveys (for information on changes in cost of health seeking behaviour)<br>2. Project records for number of referrals	All participants from the study<br><br>All referrals made in intervention and control clusters
	Indirect	Opportunity cost of increase in workload health care providers in the study area<br>Opportunity cost of the time spent by health care providers participating in the intervention's meetings	Time use survey with health workers<br><br>1.Project records on frontline workers' participation in intervention<br>2. Project records on number of health strengthening meetings and attendants<br>3. Published reports on local wage information based on skill category	A purposive sample of health workers will be selected for time use interviews<br><br>N/A
Participants/ households	Indirect	Opportunity cost of participation in the interventions, group meetings and home visits	Household survey<br>Focus group discussions<br><br>Qualitative interviews with a sub-sample of participants	All participants from the study<br>A purposive sample of 6 to 9 participants per group discussion in each of the subgroups by intervention arm<br>A purposive sample of 1 to 2 participants per each of the subgroups and intervention arms

For peer review only - http://bmjopen.bmj.com/site/about/guidelines.xhtml

Programme-related costs

Costs will be estimated using a mixed-methods approach combining costs estimated both from accounting records and using an ingredients approach (29). Resource use will be measured using financial expenditure data combined with micro-costing to allow for estimation of both financial and economic costs of each of the three intervention arms. An activity-based costing method will be applied to allocate and estimate financial costs per major program activity, using accounting records and staff time allocation sheets based on the Strengthening Economic Evaluation for Multisectoral Strategies for Nutrition (SEEMS-Nutrition) standardised tools (30). The financial cost estimates will track payments, focusing on the resources coming from organization, coordination and implementing partners including VHIR and FAS; and expenditure data for MuCCUA trial coming from Crescer project expense reports. In addition, country salary scales will be used to estimate costs incurred by implementation partners (municipal supervisors, community activities assistants and medical staff). The ingredients approach will be used to estimate economic costs, costs not included in accounting records and indirect costs incurred by beneficiaries; these will be collected via key informant interviews, focus group discussions, structured questionnaires, household surveys and/or observation (31). Furthermore, qualitative information will be collected from purposefully selected key informants from implementing organizations (VHIR, FAS), government officials (municipal supervisors, community activities assistants, FAS Coordinators, and health workers), beneficiaries and other members of the community. Focus group discussions with beneficiaries will be performed to better understand opportunity costs to them and their communities from participating in the interventions. In order to use the activity-based costing methodology, staff time allocation will be assessed to estimate and allocate costs to activities.

All costs will be collected prospectively from the project expense reports of the implementing and technical partners and entered into a programme costing tool in Microsoft Excel on an annual or monthly basis as appropriate. Data collection will be for the 3 year (2022-2025) time period from the launch of MuCCUA community trial. This method is recommended to assess the total costs of complex multi-sectoral nutrition and public health interventions that have multiple health and non-health effects (22).

For peer review only - http://bmjopen.bmj.com/site/about/guidelines.xhtml

Financial or expenditure data

Financial costs will be collected by extraction of accounting sheets provided by financial officers of implementing partners (VHIR and FAS). Data from administrative financial records will be entered in excel worksheets and assigned cost category codes for activities, inputs, thematic areas, and timing of activities by implementation partners. For capital and start-up costs occurring at the start of the project, we will adjust financial records to reflect both financial and economic costs. Data obtained from interviews will be used to estimate averages and ranges of project-related time use by each type of implementing partner, and for project beneficiaries. We will estimate the total time for implementing partner and beneficiaries time and value it with either the appropriate civil servant cost per minute (for government workers) or use a prevailing wage rate for rural or urban staff and beneficiaries. We will add in average costs of out of pocket expenses that have not been covered by the project. Labor and supply costs will then be coded (allocated) to the specific project activities and combined with the financial expenditure data to obtain total program financial and economic costs. Capital and start-up costs will be annualized over their expected useful life (32).

Donated items and opportunity costs

Some resources, such as donated items and volunteer time, are not routinely captured in project accounting records and will need to be identified and converted to economic costs using their market value (32–34). Common donated items are equipment or other capital items donated or owned by implementing agency. These will be identified through key informant interviews with the project staff.

Most of the volunteer time is related to designing messages for the Standard of care intervention, where several meetings were held with experts who were volunteers. Detailed information on the number of meetings, their duration and participants is being documented by the project. The opportunity cost of the time invested by the experts will be estimated as a proportion of their salary or a salary equivalent using published national/local wage rate reports. In addition, some community members volunteer for the project mainly in sensitization activities. The opportunity cost of volunteer time

For peer review only - http://bmjopen.bmj.com/site/about/guidelines.xhtml

donated to program implementation will be estimated using published national/local wage rate reports.

Public health provider cost/ Provider or health system cost

MuCCUA interventions are likely to increase demand for care services provided by community frontline health and nutrition workers such as ADECOS, Community Activity Assistants and midwifes, and other health care providers, such as malnutrition treatment centres or nutrition rehabilitation centres and primary health centres in the study area. In addition, there is a time cost of direct involvement in the project activities for the frontline workers and healthcare providers.

Cost of changes in utilization of health and nutrition providers

Changes in the utilisation of health and nutrition services may occur in the form of increased demand as malnutrition and other child illnesses are better identified in the communities by ADECOS. These changes in service use provided by the frontline workers and other health care providers will be collected from study participants in both intervention and control areas at the MuCCUA end-line household survey.

Opportunity costs of MuCCUA activities/ health workers

The MuCCUA interventions may increase the workload of community health workers by increasing demand for their services. In contrast, the new community-based workers (or ADECOS) introduced by the MuCCUA trial, coupled with a stronger emphasis on preventive activities; may decrease the workload of existing community health workers. In addition, project activities include sensitization and training sessions for healthcare providers in maternal, infant and young child health preventive activities in the intervention and control arms. Information on the number of meetings, their duration and participation is being documented by the project. The opportunity cost of involvement in the SOC intervention, or the value of the time spent by frontline workers will be measured as a proportion of their salary, using publicly available data on their salaries.

Participants and their household costs

For peer review only - http://bmjopen.bmj.com/site/about/guidelines.xhtml

MuCCUA interventions may influence participants and their households' costs in a number of ways. Impacts on beneficiaries and their families may include changes in household food and non-food consumption patterns and spending (regarding variety, quantity or quality of food), changes in health-seeking behaviors and associated costs as well as time cost of participation in the interventions and of attending group meetings and home visits.

Opportunity cost of participating in the interventions

Participating in the MuCCUA interventions will incur some direct and indirect costs to beneficiaries and their families. These costs may include the cost of traveling to the health posts, food and cash distributions sites as well as the opportunity cost of time spent traveling. Basic travel time and costs will be measured using survey questions to all study participants during household surveys. In addition, the cost of attending community group meetings and engaging in household visits will be estimated by collecting time allocation data from the individuals attending the group meetings at several household surveys. Focus group discussions with a sub-sample of participants purposively selected and equally distributed across the intervention and control arms, will also be undertaken to explore any other opportunity costs to households and their communities of the implementation of the interventions. All these costs will feed into the overall cost analysis.

Cost-efficiency analysis

Total costs will be estimated for each of the three arms of the MuCCUA trial: Standard of care, Standard of care + Nutrition supplementation and Standard of care + Cash transfers. We will estimate the cost-efficiency of MuCCUA trial by combining activity-specific cost data with routine data on program outputs, in order to calculate the cost per output achieved. Numbers of index pregnant women, their new born children until they are 24 months old and households reached by the intervention will be tallied by program staff. Ratios of cost per woman, child, and household reached will be calculated by dividing the total estimated intervention costs by the respective numbers reached.

Cost-effectiveness analysis

For peer review only - http://bmjopen.bmj.com/site/about/guidelines.xhtml

Economic evaluation will be conducted as a within-trial analysis using the intention-to-treat results, and will be presented in terms of ICERs, calculated as the difference in total costs of standard care only (or control) versus nutritional supplementation or cash transfer divided by the difference in mean effects of each intervention versus control. As mentioned previously, ICERs will be calculated in terms of cost per case of stunting case prevented and cost per mortality case averted among children at 24 months old.

The incremental cost-effectiveness ratio will be calculated to compare the additional costs per improved outcome achieved in interventions, including nutritional supplements or cash transfers, compared to the standard of care intervention. Where feasible we will disaggregate costs for different timeframes, including start-up vs. recurrent costs.

All costs will be presented in 2022 Angola Kwanzas (AOA) and in US Dollars (USD). We will use the average monthly exchange rate for the year of cost data collection to convert costs into USD. Any costs encountered in the past will be inflated using the local GDP deflator of Angola, before converting to USD. In addition, some expenses will be in euros and we will use the average exchange rate for the year of cost data collection to convert costs into USD. All costs will be adjusted for inflation using the Angolan Consumer Price Index and will be converted to 2022 USD using the purchasing power parity conversion factor for Angola. Moreover, the costs will be discounted using a standard discount rate of 3%, as recommended by WHO-CHOICE (35) and the Gates/IDSi Reference Case for Economic Evaluation (36).

Conclusion

The findings of this trial will provide evidence on which intervention package can have the greatest impact on preventing stunting in children under two years in Angola and improve comparability with other contexts. Robust understanding of the costs and benefits of these types of nutrition strategies is critical for priority setting and to motivate ongoing donor investment and government partnership.

The cost estimation of the different arms of the trial will complement the effectiveness results to provide useful information to the Government of Angola and other policymakers on the most cost-effective and cost-efficient intervention to prevent

For peer review only - http://bmjopen.bmj.com/site/about/guidelines.xhtml

stunting. Additionally, this study aims to gain understanding on the cost drivers, to promote inclusion of economic evaluations in other nutrition intervention studies, and to improve comparability with other contexts.

One of the limitations of the MuCCUA trial is that it is only powered to test the differences between each of the intervention arms and the comparison group, but not to test the differences between the two intervention arms. The latter would require a larger sample size and resources beyond those available for the trial. However, the possibility of a direct comparison between the interventions will be explored. The protocol has all necessary ethical approvals and the findings of this study will be disseminated within academia and the wider policy sphere.

Author affiliations

[1] Centro Nacional de Medicina Tropical, Instituto de Salud Carlos III, Avda. Monforte de Lemos, 3 Madrid 28029, Spain

[2]Tropical Medicine Unit Vall d'Hebron-Drassanes, Infectious Diseases Department, Vall d'Hebron University Hospital, PROSICS Barcelona, Barcelona 08035, Spain

[3]Centro de Investigación Biomédica en Red de Enfermedades Infecciosas, (CIBERINFEC), Instituto de Salud Carlos III, 28029 Madrid, Spain

[4]Centro Nacional de Epidemiología, Instituto de Salud Carlos III, Avda. Monforte de Lemos, 3 Madrid 28029, Spain

[5]Fundo Apoio Social-Local Development Institute,Avenida Pedro de Castro Vandunem, Luanda, Angola

[6]Faculdade de Medicina da Universidade Mandume Ya Ndemufayo, Bairro Comercial, Avenida Hoji Ya Haenda nº30, caixa postal 201, Lubango, Huíla, Angola

[7]Action Against Hunger Spain, C/Duque de Sevilla 3, Madrid 28002, Spain

[8]Program in Public Health, Stony Brook University, Stony Brook, New York, USA

Word Count: 4948

Author Contributions

RM-C and EC designed the protocol for the Economic Evaluation and prepared the first draft of the paper. ET, MR-B, ZH, IA, FR, MLF, AS-G, EI, AV, TM, AG, JCL, IM and EC contributed to the conception and design of the MuCCUA trial protocol in which this

For peer review only - http://bmjopen.bmj.com/site/about/guidelines.xhtml

study is embedded. RM-C, EC and CP designed the cost data collection tools and the processes for allocating costs. ET, IA, FR, MLF, AS-G led the supervision of all intervention implementation activities and contributed to field data acquisition. All authors contributed to the review of this manuscript and provided comments. All authors have read and approve the submitted manuscript.

Acknowledgements

We would like to acknowledge the MuCCUA study participants and field teams including ADECOS, their supervisors and students of the Universidade Mandume ya Ndemufayo for their collaboration in the data collection process. Furthermore to the Crescer consortium, municipal and province administrations of Huila and Cunene and the health and social action offices of the National Government of Angola.

Funding The Crescer project is funded by the UE EuropeAid contract nº FED/2020/418-106 and co-financed by the Crescer consortium partners. The MuCCUA trial is funded by the European Union as a part of the Crescer project (IV component of the FRESAN Program) for the 4-year duration of the project.

Data statement The datasets used and/or analysed during the current study are available from the project coordinator on reasonable request.

Competing interests None declared.

Ethics approval The research protocol was approved on August 18th, 2022 by the República de Angola Ministério da Saúde Comité de Ética (Instituto Nacional de Investigações em Saúde), reference number 27C.E/MINSA.INIS/2022. The trial was registered in clinical trials on October 7th 2022, NCT05571280.

References

1. Victora CG, Adair L, Fall C, Hallal PC, Martorell R, Richter L, et al. Maternal and child undernutrition: consequences for adult health and human capital. Lancet Lond Engl. 2008 Jan 26;371(9609):340–57.

2. de Onis M, Branca F. Childhood stunting: a global perspective. Matern Child Nutr. 2016;12(S1):12–26.

For peer review only - http://bmjopen.bmj.com/site/about/guidelines.xhtml

3. UNICEF/WHO/World Bank Group Joint Child Malnutrition Estimates. Levels and trends in child malnutrition. [Internet]. Available from: https://data.unicef.org/resources/jme-report-2021/

4. Angola Appeal [Internet]. [cited 2022 Jun 6]. Available from: https://www.unicef.org/appeals/angola

5. Angola-Humanitarian-SitRep-June-2022.pdf [Internet]. [cited 2023 Jan 20]. Available from: https://www.unicef.org/media/126971/file/Angola-Humanitarian-SitRep-June-2022.pdf

6. IPC_Angola_FoodSecurity&Nutrition_2021July2022Mar_Snapshot_English.pdf [Internet]. [cited 2022 Mar 28]. Available from: https://www.ipcinfo.org/fileadmin/user_upload/ipcinfo/docs/IPC_Angola_FoodSecurity&Nutrition_2021July2022Mar_Snapshot_English.pdf

7. WFP Angola Country Brief, September 2022 - Angola | ReliefWeb [Internet]. [cited 2023 Jan 20]. Available from: https://reliefweb.int/report/angola/wfp-angola-country-brief-september-2022

8. Governo de Angola, Unicef, World Vision, Technical Rapid Response Team. SMART Nutrition Surveys in drought-affected municipalities of Huila and Cunene provinces in Southern Angola. November-December 2019. 2020.

9. Hess SY, Peerson JM, Becquey E, Abbeddou S, Ouédraogo CT, Somé JW, et al. Differing growth responses to nutritional supplements in neighboring health districts of Burkina Faso are likely due to benefits of small-quantity lipid-based nutrient supplements (LNS). PloS One. 2017;12(8):e0181770.

10. Cooper JE, Benmarhnia T, Koski A, King NB. Cash transfer programs have differential effects on health: A review of the literature from low and middle-income countries. Soc Sci Med 1982. 2020 Jan 25;247:112806.

11. Hospital Universitari Vall d'Hebron Research Institute. Maternal and Child Interventions Against Chronic Malnutrition in Angola. A Cluster-randomized Cost-benefit Community-based Study. [Internet]. clinicaltrials.gov; 2023 Jan [cited 2023 Feb 2]. Report No.: NCT05571280. Available from: https://clinicaltrials.gov/ct2/show/NCT05571280

12. Heidkamp RA, Piwoz E, Gillespie S, Keats EC, D'Alimonte MR, Menon P, et al. Mobilising evidence, data, and resources to achieve global maternal and child undernutrition targets and the Sustainable Development Goals: an agenda for action. The Lancet. 2021 Apr 10;397(10282):1400–18.

13. Adu-Afarwuah S, Lartey A, Brown KH, Zlotkin S, Briend A, Dewey KG. Randomized comparison of 3 types of micronutrient supplements for home fortification of complementary foods in Ghana: effects on growth and motor development. Am J Clin Nutr. 2007 Aug 1;86(2):412–20.

14. Phuka JC, Maleta K, Thakwalakwa C, Cheung YB, Briend A, Manary MJ, et al. Postintervention growth of Malawian children who received 12-mo dietary complementation with a lipid-based nutrient supplement or maize-soy flour. Am J Clin Nutr. 2009 Jan 1;89(1):382–90.

For peer review only

15. Hambidge KM, Westcott JE, Garcés A, Figueroa L, Goudar SS, Dhaded SM, et al. A multicountry randomized controlled trial of comprehensive maternal nutrition supplementation initiated before conception: the Women First trial. Am J Clin Nutr. 2019 Feb 1;109(2):457–69.

16. Fenn B, Colbourn T, Dolan C, Pietzsch S, Sangrasi M, Shoham J. Impact evaluation of different cash-based intervention modalities on child and maternal nutritional status in Sindh Province, Pakistan, at 6 mo and at 1 y: A cluster randomised controlled trial. PLoS Med. 2017 May;14(5):e1002305.

17. Keats EC, Das JK, Salam RA, Lassi ZS, Imdad A, Black RE, et al. Effective interventions to address maternal and child malnutrition: an update of the evidence. Lancet Child Adolesc Health. 2021 May 1;5(5):367–84.

18. Wun J, Kemp C, Puett C, Bushnell D, Crocker J, Levin C. Measurement of benefits in economic evaluations of nutrition interventions in low- and middle-income countries: A systematic review. Matern Child Nutr. 2022;18(2):e13323.

19. Batura N, Hill Z, Haghparast-Bidgoli H, Lingam R, Colbourn T, Kim S, et al. Highlighting the evidence gap: how cost-effective are interventions to improve early childhood nutrition and development? Health Policy Plan. 2015 Jul 1;30(6):813–21.

20. Remme M, Martinez-Alvarez M, Vassall A. Cost-Effectiveness Thresholds in Global Health: Taking a Multisectoral Perspective. Value Health. 2017 Apr 1;20(4):699–704.

21. Ramponi F, Tafesse W, Griffin S. Economic evaluation of interventions to address undernutrition: a systematic review. Health Policy Plan. 2021 May 1;36(4):533–41.

22. Drummond M, Sculpher MJ, Claxton K, Stoddart GL, Torrance GW. Methods for the Economic Evaluation of Health Care Programmes. Oxford University Press; 2015. 461 p.

23. Drummond M, Stoddart G. Assessment of health producing measures across different sectors. Health Policy Amst Neth. 1995 Sep;33(3):219–31.

24. Jönsson B. Ten arguments for a societal perspective in the economic evaluation of medical innovations. Eur J Health Econ. 2009 Oct 1;10(4):357–9.

25. Byford S, Raftery J. Perspectives in economic evaluation. BMJ. 1998 May 16;316(7143):1529–30.

26. Instituto Nacional de Estatística [Internet]. [cited 2022 Jun 6]. Available from: https://www.ine.gov.ao/

27. Instituto Nacional de Estadística, Luanda-Angola. POBREZA MULTIDIMENSIONAL NOS MUNICÍPIOS DE ANGOLA [Internet]. 2019. Available from: https://www.ine.gov.ao/Arquivos/arquivosCarregados//Carregados/Publicacao_637586747742336023.pdf

28. Freedland KE, King AC, Ambrosius WT, Mayo-Wilson E, Mohr DC, Czajkowski SM, et al. The selection of comparators for randomized controlled trials of health-related behavioral interventions: recommendations of an NIH expert panel. J Clin Epidemiol. 2019 Jun;110:74–81.

For peer review only - http://bmjopen.bmj.com/site/about/guidelines.xhtml

29. Kaplan RS, Anderson SR. Time-driven activity-based costing. Harv Bus Rev. 2004 Nov;82(11):131–8, 150.

30. SEEMS-Nutrition [Internet]. [cited 2023 Jan 31]. Available from: https://sites.google.com/uw.edu/seems-nutrition/home

31. Johns B, Baltussen R, Hutubessy R. Programme costs in the economic evaluation of health interventions. Cost Eff Resour Alloc CE. 2003 Feb 26;1(1):1.

32. Batura N, Pulkki-Brännström AM, Agrawal P, Bagra A, Haghparast- Bidgoli H, Bozzani F, et al. Collecting and analysing cost data for complex public health trials: reflections on practice. Glob Health Action. 2014 Dec 1;7(1):23257.

33. Hutton G, Baltussen R. Cost valuation in resource-poor settings. Health Policy Plan. 2005 Jul 1;20(4):252–9.

34. Walker D. Cost and cost-effectiveness guidelines: which ones to use? Health Policy Plan. 2001 Mar 1;16(1):113–21.

35. World Health Organization, Baltussen RMPM, Adam T, Tan-Torres Edejer T, Hutubessy RCW, Acharya A, et al. Making choices in health : WHO guide to cost-effectiveness analysis [Internet]. World Health Organization; 2003 [cited 2022 Jun 13]. Available from: https://apps.who.int/iris/handle/10665/42699

36. Claxton KP, Revill P, Sculpher M, Wilkinson T, Cairns J, Briggs A. The Gates Reference Case for Economic Evaluation. [Internet]. The Bill and Melinda Gates Foundation; 2014. Available from: https://www.idsihealth.org/wp-content/uploads/2016/05/Gates-Reference-case-what-it-is-how-to-use-it.pdf

For peer review only

For peer review only - http://bmjopen.bmj.com/site/about/guidelines.xhtml

Reporting checklist for protocol of a clinical trial.

Based on the SPIRIT guidelines.

Instructions to authors

Complete this checklist by entering the page numbers from your manuscript where readers will find each of the items listed below.

Your article may not currently address all the items on the checklist. Please modify your text to include the missing information. If you are certain that an item does not apply, please write "n/a" and provide a short explanation.

Upload your completed checklist as an extra file when you submit to a journal.

In your methods section, say that you used the SPIRITreporting guidelines, and cite them as:

Chan A-W, Tetzlaff JM, Gøtzsche PC, Altman DG, Mann H, Berlin J, Dickersin K, Hróbjartsson A, Schulz KF, Parulekar WR, Krleža-Jerić K, Laupacis A, Moher D. SPIRIT 2013 Explanation and Elaboration: Guidance for protocols of clinical trials. BMJ. 2013;346:e7586

		Reporting Item	Page Number
**Administrative information**			
Title	#1	Descriptive title identifying the study design, population, interventions, and, if applicable, trial acronym	1

For peer review only

Trial registration	#2a	Trial identifier and registry name. If not yet registered, name of intended registry	2
Trial registration: data set	#2b	All items from the World Health Organization Trial Registration Data Set	All items addressed
Protocol version	#3	Date and version identifier	
Funding	#4	Sources and types of financial, material, and other support	21
Roles and responsibilities: contributorship	#5a	Names, affiliations, and roles of protocol contributors	1, 20-21
Roles and responsibilities: sponsor contact information	#5b	Name and contact information for the trial sponsor	1
Roles and responsibilities: sponsor and funder	#5c	Role of study sponsor and funders, if any, in study design; collection, management, analysis, and interpretation of data; writing of the report; and the decision to submit the report for publication, including whether they will have ultimate authority over any of these activities	

For peer review only

Roles and responsibilities: committees	#5d	Composition, roles, and responsibilities of the coordinating centre, steering committee, endpoint adjudication committee, data management team, and other individuals or groups overseeing the trial, if applicable (see Item 21a for data monitoring committee)	N/A
**Introduction**			
Background and rationale	#6a	Description of research question and justification for undertaking the trial, including summary of relevant studies (published and unpublished) examining benefits and harms for each intervention	3
Background and rationale: choice of comparators	#6b	Explanation for choice of comparators	5
Objectives	#7	Specific objectives or hypotheses	6
Trial design	#8	Description of trial design including type of trial (eg, parallel group, crossover, factorial, single group), allocation ratio, and framework (eg, superiority, equivalence, non-inferiority, exploratory)	4,7, 9

For peer review only

Methods: Participants, interventions, and outcomes

Study setting	#9	Description of study settings (eg, community clinic, academic hospital) and list of countries where data will be collected. Reference to where list of study sites can be obtained	8
Eligibility criteria	#10	Inclusion and exclusion criteria for participants. If applicable, eligibility criteria for study centres and individuals who will perform the interventions (eg, surgeons, psychotherapists)	9
Interventions: description	#11a	Interventions for each group with sufficient detail to allow replication, including how and when they will be administered	9-11
Interventions: modifications	#11b	Criteria for discontinuing or modifying allocated interventions for a given trial participant (eg, drug dose change in response to harms, participant request, or improving / worsening disease)	N/A to economic evaluation
Interventions: adherance	#11c	Strategies to improve adherence to intervention protocols, and any	N/A to economic evaluation

For peer review only

		procedures for monitoring adherence (eg, drug tablet return; laboratory tests)	
Interventions: concomitant care	#11d	Relevant concomitant care and interventions that are permitted or prohibited during the trial	N/A to economic evaluation
Outcomes	#12	Primary, secondary, and other outcomes, including the specific measurement variable (eg, systolic blood pressure), analysis metric (eg, change from baseline, final value, time to event), method of aggregation (eg, median, proportion), and time point for each outcome. Explanation of the clinical relevance of chosen efficacy and harm outcomes is strongly recommended	11-12
Participant timeline	#13	Time schedule of enrolment, interventions (including any run-ins and washouts), assessments, and visits for participants. A schematic diagram is highly recommended (see Figure)	9
Sample size	#14	Estimated number of participants needed to achieve study objectives and how it was determined, including clinical and statistical assumptions supporting any sample size calculations	9

For peer review only

Recruitment	#15	Strategies for achieving adequate participant enrolment to reach target sample size	N/A
**Methods: Assignment of interventions (for controlled trials)**			
Allocation: sequence generation	#16a	Method of generating the allocation sequence (eg, computer-generated random numbers), and list of any factors for stratification. To reduce predictability of a random sequence, details of any planned restriction (eg, blocking) should be provided in a separate document that is unavailable to those who enrol participants or assign interventions	Randomisation of main trial is mentioned briefly on pg 8 and main trial protocol referenced for more detail; sampling for Economic evaluation components is explained in Table 1
Allocation concealment mechanism	#16b	Mechanism of implementing the allocation sequence (eg, central telephone; sequentially numbered, opaque, sealed envelopes), describing any steps to conceal the sequence until interventions are assigned	Main trial protocol is referenced for more detail.
Allocation: implementation	#16c	Who will generate the allocation sequence, who will enrol participants, and	Main trial protocol is referenced for more detail.

For peer review only

		who will assign participants to interventions	
Blinding (masking)	#17a	Who will be blinded after assignment to interventions (eg, trial participants, care providers, outcome assessors, data analysts), and how	Main trial protocol is referenced for more detail.
Blinding (masking): emergency unblinding	#17b	If blinded, circumstances under which unblinding is permissible, and procedure for revealing a participant's allocated intervention during the trial	N/A
**Methods: Data collection, management, and analysis**			
Data collection plan	#18a	Plans for assessment and collection of outcome, baseline, and other trial data, including any related processes to promote data quality (eg, duplicate measurements, training of assessors) and a description of study instruments (eg, questionnaires, laboratory tests) along with their reliability and validity, if known. Reference to where data collection forms can be found, if not in the protocol	11-13

For peer review only

Data collection plan: retention	#18b	Plans to promote participant retention and complete follow-up, including list of any outcome data to be collected for participants who discontinue or deviate from intervention protocols	N/A
Data management	#19	Plans for data entry, coding, security, and storage, including any related processes to promote data quality (eg, double data entry; range checks for data values). Reference to where details of data management procedures can be found, if not in the protocol	Detail provided in the main trial protocol.
Statistics: outcomes	#20a	Statistical methods for analysing primary and secondary outcomes. Reference to where other details of the statistical analysis plan can be found, if not in the protocol	18-19
Statistics: additional analyses	#20b	Methods for any additional analyses (eg, subgroup and adjusted analyses)	12
Statistics: analysis population and missing data	#20c	Definition of analysis population relating to protocol non-adherence (eg, as randomised analysis), and any statistical methods to handle missing data (eg, multiple imputation)	N/A

For peer review only

Methods:

Monitoring

Data monitoring: formal committee	#21a	Composition of data monitoring committee (DMC); summary of its role and reporting structure; statement of whether it is independent from the sponsor and competing interests; and reference to where further details about its charter can be found, if not in the protocol. Alternatively, an explanation of why a DMC is not needed	N/A to economic evaluation
Data monitoring: interim analysis	#21b	Description of any interim analyses and stopping guidelines, including who will have access to these interim results and make the final decision to terminate the trial	N/A
Harms	#22	Plans for collecting, assessing, reporting, and managing solicited and spontaneously reported adverse events and other unintended effects of trial interventions or trial conduct	N/A to economic evaluation
Auditing	#23	Frequency and procedures for auditing trial conduct, if any, and whether the	N/A to economic evaluation

For peer review only

		process will be independent from investigators and the sponsor	
**Ethics and dissemination**			
Research ethics approval	#24	Plans for seeking research ethics committee / institutional review board (REC / IRB) approval	21
Protocol amendments	#25	Plans for communicating important protocol modifications (eg, changes to eligibility criteria, outcomes, analyses) to relevant parties (eg, investigators, REC / IRBs, trial participants, trial registries, journals, regulators)	N/A to economic evaluation
Consent or assent	#26a	Who will obtain informed consent or assent from potential trial participants or authorised surrogates, and how (see Item 32)	8
Consent or assent: ancillary studies	#26b	Additional consent provisions for collection and use of participant data and biological specimens in ancillary studies, if applicable	N/A
Confidentiality	#27	How personal information about potential and enrolled participants will be collected, shared, and maintained in order to protect	21

For peer review only

		confidentiality before, during, and after the trial	
Declaration of interests	#28	Financial and other competing interests for principal investigators for the overall trial and each study site	21
Data access	#29	Statement of who will have access to the final trial dataset, and disclosure of contractual agreements that limit such access for investigators	21
Ancillary and post trial care	#30	Provisions, if any, for ancillary and post-trial care, and for compensation to those who suffer harm from trial participation	N/A
Dissemination policy: trial results	#31a	Plans for investigators and sponsor to communicate trial results to participants, healthcare professionals, the public, and other relevant groups (eg, via publication, reporting in results databases, or other data sharing arrangements), including any publication restrictions	7
Dissemination policy: authorship	#31b	Authorship eligibility guidelines and any intended use of professional writers	N/A
Dissemination policy:	#31c	Plans, if any, for granting public access to the full protocol, participant-level dataset, and statistical code	N/A

For peer review only

reproducible research			

Appendices

Informed consent materials	#32	Model consent form and other related documentation given to participants and authorised surrogates	-
Biological specimens	#33	Plans for collection, laboratory evaluation, and storage of biological specimens for genetic or molecular analysis in the current trial and for future use in ancillary studies, if applicable	N/A

None The SPIRIT Explanation and Elaboration paper is distributed under the terms of the Creative Commons Attribution License CC-BY-NC. This checklist can be completed online using https://www.goodreports.org/, a tool made by the EQUATOR Network in collaboration with Penelope.ai

For peer review only

CHEERS 2022 Checklist

**Title**			
Title	1	Identify the study as an economic evaluation and specify the interventions being compared.	1
**Abstract**			
Abstract	2	Provide a structured summary that highlights context, key methods, results, and alternative analyses.	1-2
**Introduction**			
Background and objectives	3	Give the context for the study, the study question, and its practical relevance for decision making in policy or practice.	3-7
Methods			
**Health economic analysis plan**	**4**	**Indicate whether a health economic analysis plan was developed and where available.**	**7**
Study population	5	Describe characteristics of the study population (such as age range, demographics, socioeconomic, or clinical characteristics).	8
Setting and location	6	Provide relevant contextual information that may influence findings.	8
Comparators	7	Describe the interventions or strategies being compared and why chosen.	9-11
Perspective	8	State the perspective(s) adopted by the study and why chosen.	12
Time horizon	9	State the time horizon for the study and why appropriate.	9

For peer review only

(continued)

Discount rate	10	Report the discount rate(s) and reason chosen.	19
Selection of outcomes	11	Describe what outcomes were used as the measure(s) of benefit(s) and harm(s).	11-12
Measurement of outcomes	12	Describe how outcomes used to capture benefit(s) and harm(s) were measured.	11-12
Valuation of outcomes	13	Describe the population and methods used to measure and value outcomes.	11-12
Measurement and valuation of resources and costs	14	Describe how costs were valued.	12-18
Currency, price date, and conversion	15	Report the dates of the estimated resource quantities and unit costs, plus the currency and year of conversion.	19
Rationale and description of model	16	If modelling is used, describe in detail and why used. Report if the model is publicly available and where it can be accessed.	N/A
Analytics and assumptions	17	Describe any methods for analysing or statistically transforming data, any extrapolation methods, and approaches for validating any model used.	12-18
Characterising heterogeneity	18	Describe any methods used for estimating how the results of the study vary for subgroups.	12
Characterising distributional effects	19	Describe how impacts are distributed across different individuals or adjustments made to reflect priority populations.	12

For peer review only - http://bmjopen.bmj.com/site/about/guidelines.xhtml

(continued)

Characterising uncertainty	20	Describe methods to characterise any sources of uncertainty in the analysis.	19
Approach to engagement with patients and others affected by the study	21	Describe any approaches to engage patients or service recipients, the general public, communities, or stakeholders (such as clinicians or payers) in the design of the study.	7
**Results**			
Study parameters	22	Report all analytic inputs (such as values, ranges, references) including uncertainty or distributional assumptions.	18-19
Summary of main results	23	Report the mean values for the main categories of costs and outcomes of interest and summarise them in the most appropriate overall measure.	18-19
Effect of uncertainty	24	Describe how uncertainty about analytic judgments, inputs, or projections affect findings. Report the effect of choice of discount rate and time horizon, if applicable.	12
Effect of engagement with patients and others affected by the study	25	Report on any difference patient/service recipient, general public, community, or stakeholder involvement made to the approach or findings of the study	12
Discussion			

For peer review only - http://bmjopen.bmj.com/site/about/guidelines.xhtml

For peer review only

(continued)

Study findings, limitations, generalisability, and current knowledge	26	Report key findings, limitations, ethical or equity considerations not captured, and how these could affect patients, policy, or practice.	19-20
Other relevant information			
Source of funding	27	Describe how the study was funded and any role of the funder in the identification, design, conduct, and reporting of the analysis	19-20
Conflicts of interest	28	Report authors conflicts of interest according to journal or International Committee of Medical Journal Editors requirements.	21

From: Husereau D, Drummond M, Augustovski F, et al. Consolidated Health Economic Evaluation Reporting Standards 2022 (CHEERS 2022) Explanation and Elaboration: A Report of the ISPOR CHEERS II Good Practices Task Force. Value Health 2022;25. doi:10.1016/j.jval.2021.10.008

For peer review only

BMJ Open

Protocol for the economic evaluation of a three-arm community cluster randomized controlled trial to prevent chronic malnutrition in children under two years in rural Angola (MuCCUA trial)

Journal:	*BMJ Open*
Manuscript ID	bmjopen-2023-073349.R1
Article Type:	Protocol
Date Submitted by the Author:	13-Nov-2023
Complete List of Authors:	Martin-Cañavate, Rocio; Instituto de Salud Carlos III, Centro Nacional de Medicina Tropical<br>Custodio Cerezales, Estefania; Instituto de Salud Carlos III, Centro Nacional de Medicina Tropical<br>Trigo, Elena; Vall d'Hebron University Hospital, Infectious Diseases Department<br>Romay-Barja, María; Instituto de Salud Carlos III, Centro Nacional de Medicina Tropical; Centro de Investigación Biomédica en Red Enfermedades Infecciosas<br>Herrador, Zaida; Instituto de Salud Carlos III (ISCIII), Centro Nacional de Medicina Tropical<br>Aguado, Isabel; Vall d'Hebron University Hospital, Infectious Diseases Department<br>Ramirez, Ferran; Vall d'Hebron University Hospital, Infectious Diseases Department<br>Faria, Lourdes Maria; Fundo Apoio Social-Local Development Institute<br>Silva-Gerardo, Ana; Faculdade de Medicina da Universidade Mandume Ya Ndemufayo<br>Lima, Jose Carlos; Faculdade de Medicina da Universidade Mandume Ya Ndemufayo<br>Iráizoz, Eva; Action Against Hunger Spain<br>Marques, Tayná; Vall d'Hebron University Hospital, Infectious Diseases Department<br>Vargas, Antonio; Action Against Hunger Spain<br>Gomez, Amador; Action Against Hunger Spain<br>Puett, Chloe; Stony Brook University Program in Public Health<br>Molina, Israel; Vall d'Hebron University Hospital, Infectious Diseases Department; Centro de Investigación Biomédica en Red Enfermedades Infecciosas
**Primary Subject Heading**:	Public health
Secondary Subject Heading:	Nutrition and metabolism, Global health
Keywords:	HEALTH ECONOMICS, Nutrition < TROPICAL MEDICINE, NUTRITION & DIETETICS, EPIDEMIOLOGIC STUDIES, Community child health < PAEDIATRICS, PUBLIC HEALTH

SCHOLARONE™
Manuscripts

Protocol for the economic evaluation of a three-arm community cluster randomized controlled trial to prevent chronic malnutrition in children under two years in rural Angola (MuCCUA trial)

Rocio Martin-Cañavate[1], Elena Trigo[2], Maria Romay-Barja[1,3], Zaida Herrador[4], Isabel Aguado[2], Ferran Ramirez[2], Lourdes Maria Faria[5], Ana Silva-Gerardo[6], Jose Carlos Lima[6], Eva Iráizoz[7], Tayna Marques[2], Antonio Vargas[7], Amador Gomez[7], Chloe Puett[8], Israel Molina[2,3], Estefania Custodio[1,3*]

Correspondence to Estefanía Custodio ecustodio@isciii.es, +34 622859128, Centro Nacional de Medicina Tropical, Instituto de Salud Carlos III, Avda. Monforte de Lemos, 3 Madrid 28029, Spain

Rocio Martin-Cañavate rocio.mcanavate@isciii.es

Elena Trigo elena.trigo@vhir.org

Maria Romay-Barja mromay@isciii.es

Zaida Herrador zherrador@isciii.es

Isabel Aguado Lobo isabel.aguado@vhir.org

Ferran Ramírez ferran.ramirez@vhir.org

Lourdes Maria Faria Lourdesfaria2003@yahoo.com.br

Ana Silva-Gerardo anasgerardo@yahoo.com.br

Jose Carlos Lima rahita5y@yahoo.com.br

Eva Iráizoz Domínguez eiraizoz@accioncontraelhambre.org

Tayná Marques tayna.marques@vhir.org

Antonio Vargas avargas@accioncontraelhambre.org

Amador Gómez agomez@accioncontraelhambre.org

Chloe Puett chloepuett@gmail.com

Israel Molina israel.molina@vallhebron.cat

Estefania Custodio ecustodio@isciii.es

Abstract

Introduction: Chronic malnutrition is a serious problem in southern Angola with a prevalence of 49.9% and 37.2% in the provinces of Huila and Cunene respectively. The MuCCUA trial is a community-based randomized controlled trial (c-RCT) which aims to

For peer review only

For peer review only - http://bmjopen.bmj.com/site/about/guidelines.xhtml

evaluate the effectiveness of a nutrition supplementation plus standard of care intervention and a cash transfer plus standard of care intervention in preventing stunting, and to compare them to a standard of care alone intervention in southern Angola. This protocol describes the planned economic evaluation associated with the c-RCT.

Methods and analysis: We will conduct a cost-efficiency and cost-effectiveness analysis nested within the MuCCUA trial with a societal perspective, measuring programme, provider, participant and household costs. We will collect programme costs prospectively using a combined calculation method including quantitative and qualitative data. Financial costs will be estimated by applying activity-based costing methods to accounting records using time allocation sheets. We will estimate costs not included in accounting records by the ingredients approach, and indirect costs incurred by beneficiaries through interviews, household surveys and focus group discussions. Cost-efficiency will be estimated as cost per output achieved by combining activity-specific cost data with routine data on program outputs. Cost-effectiveness will be assessed as cost per stunting case prevented. We will calculate incremental cost-effectiveness ratios (ICER) comparing the additional cost per improved outcome of the different intervention arms and the standard of care. We will perform ensitivity analyses to assess robustness of results.

Ethics and dissemination: This economic evaluation will provide useful information to the Angolan Government and other policymakers on the most cost-effective intervention to prevent stunting in this and other comparable contexts. The protocol was approved by the República de Angola Ministério da Saúde Comité de Ética (27C.E/MINSA.INIS/2022). The findings of this study will be disseminated within academia and the wider policy sphere.

Trial registration number: NCT05571280; pre-results

Keywords: Cost-effectiveness, Stunting, Angola, Chronic malnutrition, multi sectoral nutrition programme, cash transfers, nutritional supplementation

Article Summary

For peer review only - http://bmjopen.bmj.com/site/about/guidelines.xhtml

Strengths and limitations of this study

- This protocol will contribute to the growing evidence regarding cost-effective and cost-efficient multisectoral interventions to prevent chronic malnutrition.
- The study design draws on a comprehensive and broad cost analysis based on diverse data sources such as trial data, community-level surveys, staff interviews, interviews and focus group discussions with participants, and detailed program expenditures.
- The economic evaluation design will allow societal, participant, household and provider costs to be disaggregated.
- Community-based trials pose methodological challenges in terms of data collection and participant follow-up especially in these rural and remote settings of Southern Angola.
- The economic evaluation will be conducted in the context of a research project, and costs could be influenced by the research component in ways that cannot be seen or corrected for.

BACKGROUND

Burden of child undernutrition

Stunting or chronic malnutrition is the impairment of growth and development that children experience due to malnutrition, repeated infections, and inadequate psychosocial stimulation (1). Stunting in early life, particularly in the first 1000 days, from conception through the first two years of life, has adverse physical and functional consequences in the child including higher risk of morbidity and mortality, lower educational attainment and cognition, lower adult earnings, loss of productivity, and, when accompanied by excessive weight gain in childhood, increased risk of nutrition-related chronic diseases in adulthood (2). Stunting affects 149.2 million children under 5 years of age. The number of children with stunting is declining in all regions except Africa (3).

In Angola, the level of chronic malnutrition is high, with 38% of children being stunted; and 5% of children being acutely malnourished nationally (DHS 2016-2017)(3).

For peer review only - http://bmjopen.bmj.com/site/about/guidelines.xhtml

According to UNICEF, 3.8 million people in Angola face food and nutrition insecurity due to climate shocks (4). Recurrent drought, reduced agricultural and livestock production, crop failures, high food prices and the proliferation of infectious diseases all contribute to compromising maternal and infant nutrition and health (5) (6). The South-Western provinces of Angola have been most severely affected. In the provinces of Cunene and Huila, the latest Integrated Food Security Phase Classification (IPC) analysis revealed that over 1.3 million people are estimated to be experiencing high levels of acute food insecurity with more than 50% of their total population in *Crisis* or *Emergency* levels between April 2021 and March 2022 (6). The situation has deteriorated during the lean season as food reserves were depleted, drought conditions continue to be exacerbated by erratic below-average rainfall and the humanitarian response has proven inadequate to address the most urgent needs (7). Further, a Standardised Monitoring and Assessment of Relief and Transitions (SMART) survey conducted in 2019 in selected municipalities of Huila and Cunene reported a prevalence of global acute malnutrition of 11% in both provinces, and a stunting prevalence of 49.9% and 37.2% in the provinces of Huila and Cunene respectively (8).

Due to the severity of the situation, there is a need to improve the nutritional status of this population. In relation to stunting, there is growing evidence that nutrition specific interventions such as small-quantity lipid-based nutrient supplementation (SQ-LNS) have benefits on child growth (9). The positive effects of SQ-LNS on growth was validated by a meta-analysis that provided evidence of this effect at reducing stunting or wasting in a variety of contexts, recommending to policy makers its inclusion in intervention packages (10). There is also evidence that nutrition-sensitive interventions, e.g., cash transfers, could improve nutrition and health outcomes and accelerate reductions in stunting (11)(12). The Crescer project aims to address stunting in Southern Angola by implementing a multisectoral approach with both a nutrition-specific and a nutrition-sensitive intervention.

MuCCUA trial

The Crescer project is an operational research program on the prevention of chronic malnutrition in the southern Angolan provinces of Huila and Cunene. It is embedded in the FRESAN (Fortalecimento da Resiliência e da Segurança Alimentar e Nutricional em

For peer review only - http://bmjopen.bmj.com/site/about/guidelines.xhtml

Angola) program, funded by the European Union (FED – European Development Funds) to strengthen resilience and food and nutrition security in South Angola. The Crescer project implements the MuCCUA (Mother and Child Chronic Undernutrition in Angola) trial, a three-arm community-based cluster randomized controlled trial of non-inferiority which aims to evaluate the effectiveness and cost-effectiveness of a nutrition supplementation plus standard of care intervention and a cash transfer plus standard of care intervention compared to a standard of care alone intervention in preventing stunting in children at 24 months in Huila and Cunene. Pregnant women and their newborn children are target recipients of either arm: the comparator arm receives standard of care alone, the second arm receives standard of care plus nutrition supplementation with SQ-LNS and a family food ration, and the third arm receives standard of care plus unconditional cash transfers. Community and Health Development Agents (ADECOS) implement the interventions following Angolan government protocols (13). The primary outcome is prevalence of stunting in children. The MuCCUA trial is described in detail elsewhere (14). The purpose of this protocol paper is to fully describe the methodology for the economic evaluation of the trial.

Economic evaluations of multi-sectoral interventions to prevent child stunting

Delivering multi-sectoral interventions combining nutrition-specific and nutrition-sensitive interventions during the first 1000 days of life is key to prevent chronic malnutrition and to improve early child growth (15). Reduction of stunting requires improvements in food and nutrition security, education, WASH (water, sanitation and hygiene interventions), health, the status of women and poverty reduction (2). Maternal and child interventions such as nutritional supplementation with small quantity lipid-based nutrient supplements (10,16–18), cash transfers (12,19,20), vitamin A supplementation and behaviour change communication have shown promising results in preventing chronic malnutrition (21,22). However, evidence on the effectiveness of combining them in multisectoral programs is scarce (23).

In order to advocate for further investment on these multisectoral programs there is a need to provide knowledge on their effectiveness but also on their costs (24), as funding agencies are increasingly requesting implementers to include economic evaluations in their proposals.

For peer review only - http://bmjopen.bmj.com/site/about/guidelines.xhtml

The body of economic evidence supporting nutrition programs is growing (15), but it is still limited specially for low-and middle-income countries (LMICs) (25,26) and for multisectoral nutrition interventions (15).

Multi-sectoral programs including health, nutrition, cash, and behaviour change communication interventions pose challenges for economic evaluations due to their heterogeneity. First, studies with interventions delivered in different sectors can be difficult to compare due to differences in the outcomes and costs included (27–30). Second, the perspective chosen for the economic evaluation poses another challenge for interpretation and comparison of findings between studies because of the different sets of costs included (25,31,32). And third, economic evaluation objectives differ greatly between studies as some aim to prioritize policies and funds, and different studies may aim to inform different decision makers (28).

There are published studies on the costs of complex interventions including nutrition supplementation (33) (34) or cash transfer interventions (35) (11) , but, to the best of our knowledge, there are no studies comparing the costs of these two interventions within the same project, as this study does..

Finally, a recent review on costs analysis of nutrition interventions on LIMCs highlighted the need to standardize methods and reporting in economic evaluations in order to facilitate interpretation and provide a means for comparing costs and cost-effectiveness of interventions (36). The initiative SEEMS-Nutrition (Strengthening Economic Evaluation for Multisectoral Strategies for Nutrition) has developed a common approach guide to measure the costs and benefits of multisector nutrition strategies (37), that we have followed.

The proposed economic evaluation of the MuCCUA trial aims to address the challenges mentioned above by assessing the cost-efficiency and cost-effectiveness of the interventions following the SEEMS guide. The perspective used will be societal including all costs of the interventions regardless of who incurs them.

Thus, we hope to contribute to the scarce literature on the cost-efficiency and the cost-effectiveness of implementing multisectoral nutrition interventions including both

For peer review only - http://bmjopen.bmj.com/site/about/guidelines.xhtml

nutrition specific and nutrition sensitive interventions, with a standardized methodology in a LMIC real world setting located in Southern Angola.

Aim and objectives

The aim of the MuCCUA trial economic evaluation is to use a societal perspective to estimate the costs, and evaluate the cost-efficiency and cost-effectiveness of three interventions (standard of care alone, standard of care plus nutritional supplements, and standard of care plus cash transfers) in reducing stunting in children under 2 years of age from poor and very poor households in the southern provinces of Cunene and Huila in Angola.

The specific objectives of the economic evaluation are:

1. To estimate the costs of setting up and implementing each of the MuCCUA interventions.
2. To calculate costs incurred by the intervention participants and their families as a result of the interventions.
3. To estimate the cost-efficiency of each of the three interventions as cost per woman, child, and household reached.
4. To estimate the cost-effectiveness as cost per case of stunting averted by comparing incremental costs and incremental changes in the outcomes of the interventions, compared with the standard of care.

In addition to the above objectives, heterogeneity analysis of the MuCCUA interventions will be assessed to evaluate how the costs and consequences of the interventions are distributed among different sub-groups of the study population.

METHODS

Study design

A cost-efficiency and cost-effectiveness analysis of the MuCCUA trial, a community-based cluster-randomised trial that compares three interventions to prevent children's stunting taking place between October 2022 and February 2025. We will estimate the

For peer review only - http://bmjopen.bmj.com/site/about/guidelines.xhtml

total and incremental costs of the interventions prospectively from a societal perspective, measuring programme, provider, participants and household costs.

Patient and Public involvement

Community leaders (locally named by “sobas”) and the community were involved at the early stages of the research with discussions about the outcomes and the interventions. Then ADECOS were selected by their own communities only after fulfilling certain technical and capacities criteria. Sobas, communities and ADECOS were involved in the cluster randomization. Methods of recruitment were informed by discussions with Community Activity Assistants and ADECOS through a focus group session. All women from selected randomized communities included were identified and informed of the study by ADECOS.

Pregnant women and their families were not involved in setting the research question or the outcome measures. The project is promoting understanding and dissemination of key messages around the interventions to motivate community involvement during and beyond the study. A feedback/suggestions mechanism for participants and communities has been implemented as well.

At the end of the study, results will be shared and discussed with participants, their families and communities through dedicated workshops. Results and all the knowledge generated should promote change at community, regional and national level in policies and practices.

Study setting and population

The MuCCUA trial is set in four communes of Huila and Cunene, two provinces of South Angola. These provinces are predominantly rural with a combined total population of around 4.4 million (Huila population of 3.185.244 and Cunene population of 1.271.638) (38). There are province-specific differences related to livelihoods. Huila has a higher agricultural productivity and lower food insecurity risk as compared to Cunene, mostly livestock activity, where the semi-arid conditions and frequent droughts occasionally result in significant livestock loss, a key livelihood asset. Overall, the population is highly dependent on staple foods (primarily maize, millet and sorghum) and there is poor access to safe drinking water, hygiene and sanitation which may differ by commune also,

For peer review only - http://bmjopen.bmj.com/site/about/guidelines.xhtml

For peer review only

due to the communes' different geographical features. The socioeconomic status of clusters and households is expected to be homogeneous, as one of the clusters inclusion criteria was to experience extreme poverty. Different ethnic groups are present in the clusters but we do not expect diverse access to interventions according to ethnic origin as none of this groups are nomad. However, access to health care and other essential services differs in relation to the distance from the communities to the main settlements of each of the communes. There are remote communities where long distance, lack of transport and lack of trained health professionals and medicines, makes access to basic services inadequate.

The study participants are pregnant women confirmed by pregnancy test, over 16 years of age, and their new-born children. The target population of the interventions is the household in which the pregnant woman and their children live. In the case of having more than one pregnant woman within the household, all women who accept to participate will be included. We consider households to be the person or group of persons, with or without kinship relationships, who have been habitually living under the same roof for at least 6 months or less but with the intention of staying in the residence for the next 6 months and share food and/or other vital needs such as water, cooking and eating utensils and hygiene products. Informed cluster-level consent was sought from village leaders, Sobas, to participate in the trial before randomization. After acceptance to participate in the study through the informed consent, mothers and their children are followed up from the time of pregnancy at the time of recruitment until the child is 24 months old. .

Trial design

In the MuCCUA trial, a total of 36 purposively-selected clusters (the selection of which is described below), with an estimated population of around 55000, were randomized to the three trial arms with 6 clusters per arm in Huíla province, and 6 clusters per arm in Cunene province. Two municipalities (admin 2) were selected per province (admin1) with one commune (admin 3) per municipality (total 2 communes per province). In the province of Huíla, the commune of Libongue (municipality of Chicomba) and the commune of Jamba Sede (municipality of Jamba) were selected. In the province of

For peer review only - http://bmjopen.bmj.com/site/about/guidelines.xhtml

Cunene, the commune of Otchinjau (municipality of Cahama) and the commune of Mupa-Mukolongodjo (municipality of Cuvelai) were selected. The cluster unit is typically a village or neighbourhood and any nearby hamlets within each commune or, in the case of large neighbourhoods, a distinct part of it that is similar in population size to the unit. The randomisation, which took place in September 2022, was stratified by cluster.
The inclusion criteria for clusters was being considered as one of the municipalities prioritized by the FRESAN program and having a multidimensional poverty level 4 or 5 according to the *Instituto Nacional de Estatística* classification (39), not currently having or planned to have in operation other interventions offering monetary or nutritional transfers, acceptance by the municipal and traditional authorities, accessible in a 4x4 vehicle and having a reference health post with reference health personnel.

Recruitment and baseline survey started in October 2022 and will continue until March 2023. Implementation started at recruitment and is planned to end in November 2025. A total of 1440 pregnant women and their newborns, 480 in each arm are enrolled. Primary efficacy analyses will be performed when participant's children have completed a total of 24 months of follow-up. A full description of the intervention design within the MuCCUA trial is described in detail elsewhere (14). A brief description of the interventions is presented in the following sections.

MuCCUA interventions

Standard of care arm (SOC)

This is the comparator arm. The communities allocated to this arm receive as intervention the standard of care alone, as described below.

The standard of care intervention comprises the recruitment of ADECOS (Community and Health Development Agents) and the implementation of a set of activities described in the National Development Plan of Angola (40) and in the Multisectoral Strategic Plan on Nutrition (41), aligned with by WHO maternal and infant health care recommendations, following the National Policy of ADECOS (PNADECOS). This intervention will be used as a comparator as it fulfils the criteria of acceptability,

For peer review only - http://bmjopen.bmj.com/site/about/guidelines.xhtml

feasibility, relevance and uniformity required to be a good comparator in this type of studies (42). The standard of care activities can be grouped into two blocks.

1) Health promotion activities: including community interventions to promote adequate infant and young child feeding (IYCF) practices, identification of danger signs of malnutrition and referral to health posts and promotion of adequate hygiene, water and sanitation practices through sensitization activities through the figure of ADECOS.

2) Preventive pharmacological activities: including semiannual vitamin A supplementation for children 6-24 months old, semiannual deworming with albendazole for children from 12 months to 24 months of age and pregnant women from the 2nd trimester; and malaria prophylaxis with sulfadoxine-pyrimethamine for pregnant women from 13th week of pregnancy through the health posts.

The standard of care is implemented in all the communities participating in the study, as detailed below..

Standard of care + Nutritional supplementation (SOC + NUT)

Communities allocated to this arm will receive the standard of care intervention plus nutritional supplementation to families with at least one pregnant woman enrolled. Nutritional supplementation will consist of SQ-LNS, a peanut-based ready-to-use home-fortification product to improve diet quality, for the pregnant women and their newborn children, and a complementary food ration for their families. The rations will be as follows: an individual ration of SQ-LNS for pregnant women Enov'Mum (1 sachet of 20 g daily) and Enov'Nutributter for children over 6 months (1 sachet of 20 g daily) produced and packaged in France by Nutriset®. A complementary family food ration will comprise of a basket of locally produced staple foods that complements the usual diet (300 Kcal/person/day). The caloric distribution of the basket will be 45% of cereals (corn meal - carbohydrate), 30% of legumes (beans - vegetable protein) and 25% of oil (soybean oil - fat). In addition, 1 kg of iodized salt will be provided. The food basket is distributed in order to improve adherence to the SQ-LNS, contribute to greater participation and prevent intra-family distribution of individual rations.

Standard of care + Cash transfer (SOC + CT)

For peer review only - http://bmjopen.bmj.com/site/about/guidelines.xhtml

Communities allocated to this arm will receive the standard of care intervention plus an unconditional cash transfer to families with at least one pregnant woman enrolled. A total of 13855 kwanzas/month (US$31.5, Oct22) will be delivered to households with 4 or more inhabitants. A total of 10855 kwanzas/month (US$24.7, Oct22) will be delivered to households with 3 or fewer inhabitants. The minimum inter professional salary in Angola at the time being is set at 35000 kwanzas per month (US$79.5, Oct22). The amount will be directly delivered to study participants. The amounts of cash were selected based on the amount disbursed by the *Kwenda* project, an Angolan government program funded by the World Bank aiming to fight poverty and to promote sustainable development in the communities with very poor households.

Measurement of health outcomes/effectiveness

The outcomes of the interventions will be assessed through repeated cross-sectional household surveys at baseline, at 6 months of pregnancy, 9 months of pregnancy, 3, 6, 12, 18 months of the newborn child, and end-line at 24 months of newborn child.

The MuCCUA trial will test the effect of each of the experimental interventions (SOC + NUT, SOC + CT arms) relative to the control (SOC arm). A detailed description of the trial outcomes is included in the trial protocol (14).

Primary outcome

The MuCCUA trial primary outcome is prevalence of stunting as measured by height-for-age z-score (HAZ) or length-for-height (LAZ) < − 2 standard deviations (SD) and mean HAZ or LAZ score in children at 24 months of age in the provinces of Huila and Cunene.

The trial sample size was powered to measure an expected 13% reduction in stunting overall and to detect a difference in the non-inferiority margin of 10 % between the proportions in the intervention groups (SOC + NUT and SOC + CT) and the comparison group (SOC alone) post intervention. We will conduct a cost-effectiveness analysis for cases of stunting averted if there is evidence of impact on the prevalence of stunting in children at 24 months at the 0.1 significance level or below. Cases averted by the two interventions relative to the control group for the outcome will be calculated at 24 months.

For peer review only - http://bmjopen.bmj.com/site/about/guidelines.xhtml

Secondary outcomes

The MuCCUA trial has a number of secondary outcomes including: infant mortality rate at 3, 6 , 12, 18 and 24 months, proportion of neonatal low birth weight, and low birth weight for gestational age, proportion of children with anaemia, cumulative incidence of morbidity (malaria, diarrhoea and pneumonia), primary caregiver's knowledge, attitudes and practices related to perinatal and children's caring practices including breastfeeding and hygiene and sanitation, and women and children minimum dietary diversity.

Heterogeneity analyses

In addition to calculating the efficiency and cost-effectiveness of the MuCCUA trial, heterogeneity analysis will be conducted to assess how costs and impacts of the interventions are distributed among the target population. The economic evaluation will be stratified through subgroup analyses of the primary outcome based on differences of the study population characteristics. Disaggregation of the findings will be performed in subgroups per treatment arm, based on the two provinces and on the four communes of the study. The environment and way of living in each of the two provinces are slightly different in terms of for example distances between household's compounds and to health services. In the province of Cunene, households, are very far apart from each other and they rely solely on themselves not having much interaction with other members of the communes. On the contrary, in the province of Huila, communities live closer together in villages and there more interactions exist between them. Socioeconomic status will not be included as a subgroup as we do not expect important differences because one of the inclusion criteria to participate in the study was to experience extreme poverty. In each of these subgroups community group discussions will be carried out with different samples of participants according to their access to basic services and distance to the distribution points of the interventions.

Identification, measurement and valuation of resource use

The cost-efficiency and cost-effectiveness of the MuCCUA interventions will be measured from a societal perspective (31,32); taking into account costs incurred by the program providers, namely the Institute of Local Development-Social Support Fund of

For peer review only - http://bmjopen.bmj.com/site/about/guidelines.xhtml
For peer review only

Angola (FAS) and Vall d'Hebron Institute of Research (VHIR) (programme-provider costs), ADECOS and by the beneficiaries who are intervention participants and their households. Resource use will be measured using financial expenditure data combined with micro-costing to allow for estimation of both financial and economic costs. All cost data will be collected prospectively from the launch of the MuCCUA community trial project for a 3-year (2022-2025) time period, and divided into start-up and recurrent costs. Table 1 provides an overview of the resource use and cost measures that are described in more detail further below.

For peer review only

For peer review only - http://bmjopen.bmj.com/site/about/guidelines.xhtml

Table 1 Overview of resource use and cost measures included in the economic evaluation of the MuCCUA interventions

**Perspective/cost category**	**Type of costs**	**Description**	**Source**	**Sample size**
Programme/ implementing agency	Direct	Costs of implementing the intervention	1. Project accounts of the implementing agencies<br>2. Interviews with the project staff	N/A
	Indirect	Opportunity cost of donated items, volunteer time	1. Interviews with the project staff<br>2. Project records on volunteer involvement<br>3. Project accounts	N/A
Provider/ health system o (Health providers)	Direct	Changes in demand/ utilization of nutrition and health services / Costs of referrals	1. Baseline and endline cross-sectional surveys (for information on changes in cost of health seeking behaviour)<br>2. Project records for number of referrals	All participants from the study<br><br>All referrals made in intervention and control clusters
	Indirect	Opportunity cost of increase in workload health workers in the study area<br>Opportunity cost of the time spent by health workers participating in the intervention's meetings	Time use survey with health workers<br><br>1.Project records on health workers' participation in intervention<br>2. Project records on number of health strengthening meetings and attendants<br>3. Published reports on local wage information based on skill category	A purposive sample of health workers will be selected for time use interviews<br><br>N/A
Participants/ households	Indirect	Opportunity cost of participation in the interventions, group meetings and home visits	Household survey<br>Focus group discussions<br><br>Qualitative interviews with a sub-sample of participants	All participants from the study<br>A purposive sample of 6 to 9 participants per group discussion in each of the subgroups by intervention arm<br>A purposive sample of 1 to 2 participants per each of the subgroups and intervention arms

For peer review only - http://bmjopen.bmj.com/site/about/guidelines.xhtml

Programme-related costs

Costs will be estimated using a mixed-methods approach combining costs estimated both from accounting records and using an ingredients approach (43). Resource use will be measured using financial expenditure data combined with micro-costing to allow for estimation of both financial and economic costs of each of the three intervention arms. An activity-based costing method will be applied to allocate and estimate financial costs per major program activity, using accounting records and staff time allocation sheets based on the Strengthening Economic Evaluation for Multisectoral Strategies for Nutrition (SEEMS-Nutrition) standardised tools (37). The financial cost estimates will track payments, focusing on the resources coming from organization, coordination and implementing partners including VHIR and FAS; and expenditure data for MuCCUA trial coming from Crescer project expense reports. In addition, country salary scales will be used to estimate costs incurred by implementation partners (municipal supervisors, community activities assistants and medical staff). The ingredients approach will be used to estimate economic costs, costs not included in accounting records and indirect costs incurred by beneficiaries; these will be collected via key informant interviews, focus group discussions, structured questionnaires, household surveys and/or observation (44). Qualitative information will be collected through interviews from purposefully selected key informants from implementing organizations (VHIR, FAS), government officials (municipal supervisors, community activities assistants, FAS coordinators, and health workers), beneficiaries and other members of the community. In order to use the activity-based costing methodology, staff time allocation will be assessed to estimate and allocate costs to activities. In addition, focus group discussions with beneficiaries will be performed to better understand opportunity costs to them and their communities from participating in the interventions. A total of 24 focus groups discussion including 8 to 10 beneficiaries will be performed, two per trial arm in each of the four communes.

All costs will be collected prospectively from the project expense reports of the implementing and technical partners and entered into a programme costing tool in Microsoft Excel on an annual or monthly basis as appropriate. Data collection will be for the 3 year (2022-2025) time period from the launch of MuCCUA community trial. This

For peer review only - http://bmjopen.bmj.com/site/about/guidelines.xhtml

method is recommended to assess the total costs of complex multi-sectoral nutrition and public health interventions that have multiple health and non-health effects (29).

Financial or expenditure data

Financial costs will be collected by extraction of accounting sheets provided by financial officers of implementing partners (VHIR and FAS). Data from administrative financial records will be entered in excel worksheets and assigned cost category codes for activities, inputs, thematic areas, and timing of activities by implementation partners. For capital and start-up costs occurring at the start of the project, we will adjust financial records to reflect both financial and economic costs. Data obtained from interviews will be used to estimate averages and ranges of project-related time use by each type of implementing partner, and for project beneficiaries. We will estimate the total time for implementing partner and beneficiaries time and value it with either the appropriate civil servant cost per minute (for government workers) or use a prevailing wage rate for rural or urban staff and beneficiaries. We will add in average costs of out of pocket expenses that have not been covered by the project. Labor and supply costs will then be coded (allocated) to the specific project activities and combined with the financial expenditure data to obtain total program financial and economic costs. Capital and start-up costs will be annualized over their expected useful life (45).

Donated items and opportunity costs

Some resources, such as donated items and volunteer time, are not routinely captured in project accounting records and will need to be identified and converted to economic costs using their market value (45–47). Common donated items are equipment or other capital items donated or owned by implementing agency. These will be identified through key informant interviews with the project staff.

Most of the volunteer time is related to designing messages for the Standard of care intervention, where several meetings were held with experts who were volunteers. Detailed information on the number of meetings, their duration and participants is being documented by the project. The opportunity cost of the time invested by the experts will be estimated as a proportion of their salary or a salary equivalent using published national/local wage rate reports. In addition, some community members volunteer for

For peer review only - http://bmjopen.bmj.com/site/about/guidelines.xhtml

the project mainly in sensitization activities. The opportunity cost of volunteer time donated to program implementation will be estimated using published national/local wage rate reports.

Public health provider cost/ Provider or health system cost

MuCCUA interventions are likely to increase demand for care services provided by community frontline health and nutrition workers such as ADECOS, Community Activity Assistants and midwifes, and other health care providers, such as malnutrition treatment centres or nutrition rehabilitation centres and primary health structures in the study area. In addition, there is a time cost of direct involvement in the project activities for the frontline workers and healthcare providers.

Cost of changes in utilization of health and nutrition providers

Changes in the utilisation of health and nutrition services may occur in the form of increased demand as risk of malnutrition and other child illnesses are better identified in the communities by ADECOS. These changes in service provided by health care providers will be collected through questionnaires administered from study participants in both intervention and control areas at the MuCCUA end-line household survey. The questionnaires ask participants on the frequency of attendance to the health posts, at baseline and at end line and results will be compared before and after, and between arms.

Opportunity costs of MuCCUA activities/health workers

The MuCCUA interventions may increase the workload of health workers by increasing demand for their services. In contrast, the new community-based workers (or ADECOS) introduced by the MuCCUA trial, coupled with a stronger emphasis on preventive activities; may decrease the workload of existing health workers in the study areas. In addition, project activities include sensitization and training sessions for healthcare providers in maternal, infant and young child health preventive activities in the intervention and control arms. Information on the number of meetings, their duration and participation is being documented by the project. The opportunity cost of involvement in the SOC intervention, or the value of the time spent in training by health

For peer review only - http://bmjopen.bmj.com/site/about/guidelines.xhtml

workers will be measured as a proportion of their salary, using publicly available data on their salaries.

Participants and their household costs

MuCCUA interventions may influence participants and their households' costs in a number of ways. Impacts on beneficiaries and their families may include changes in household food and non-food consumption patterns and spending (regarding variety, quantity or quality of food), changes in health-seeking behaviors and associated costs as well as time cost of participation in the interventions and of attending group meetings and home visits.

Opportunity cost of participating in the interventions

Participating in the MuCCUA interventions will incur some direct and indirect costs to beneficiaries and their families. These costs may include the cost of traveling to the health posts, food and cash distributions sites as well as the opportunity cost of time spent traveling. Basic travel time and costs will be measured using survey questions to all study participants during household surveys. In addition, the cost of attending community group meetings and engaging in household visits performed by the ADECOS will be estimated by collecting time allocation data from the individuals attending the group meetings at several household surveys. Focus group discussions with a sub-sample of participants purposively selected and equally distributed across the intervention and control arms, will also be undertaken to explore any other opportunity costs to households and their communities of the implementation of the interventions. All these costs will feed into the overall cost analysis.

Cost-efficiency analysis

Total costs will be estimated for each of the three arms of the MuCCUA trial: Standard of care, Standard of care + Nutrition supplementation and Standard of care + Cash transfers. We will estimate the cost-efficiency of MuCCUA trial by combining activity-specific cost data with routine data on program outputs, in order to calculate the cost per output achieved. Numbers of index pregnant women, their new born children until they are 24 months old and households reached by the intervention will be tallied by

For peer review only - http://bmjopen.bmj.com/site/about/guidelines.xhtml

program staff. Ratios of cost per woman, child, and household reached will be calculated by dividing the total estimated intervention costs by the respective numbers reached.

Cost-effectiveness analysis

Economic evaluation will be conducted as a within-trial analysis using the intention-to-treat results, and will be presented in terms of ICERs, calculated as the difference in total costs of standard care only (or comparator) versus nutritional supplementation or cash transfer divided by the difference in mean effects of each intervention versus control. As mentioned previously, ICERs will be calculated in terms of cost per stunting case prevented among children at 24 months old if there is a statistically significant difference in the outcome between intervention study arms.

The incremental cost-effectiveness ratio will be calculated to compare the additional costs per improved outcome achieved in interventions, including nutritional supplements or cash transfers, compared to the standard of care intervention. Where feasible we will disaggregate costs for different timeframes, including start-up vs. recurrent costs.

Sensitivity analysis will be performed to determine the extent to which the results of the analysis might change given plausible variation in study parameters related to costs drivers and stunting rates. To obtain an estimate of how costs could vary, a range of costs will be calculated based on different scenarios such as local costs scenario, products price or coverage. Univariate and multivariate probabilistic sensitivity analyses will assess effect of the main cost drivers on the results.

All costs will be presented in 2022 Angola Kwanzas (AOA) and in US Dollars (USD). We will use the average monthly exchange rate for the year of cost data collection to convert costs into USD. Any costs encountered in the past will be inflated using the local gross domestic product deflator of Angola, before converting to USD. In addition, some expenses will be in euros and we will use the average exchange rate for the year of cost data collection to convert costs into USD. All costs will be adjusted for inflation using the Angolan Consumer Price Index and will be converted to 2022 USD using the purchasing power parity conversion factor for Angola. Moreover, the costs will be discounted using

For peer review only - http://bmjopen.bmj.com/site/about/guidelines.xhtml

a standard discount rate of 3%, as recommended by WHO-CHOICE (48) and the Gates/IDSi Reference Case for Economic Evaluation (49).

Conclusion

The findings of this trial will provide evidence on which intervention package can have the greatest impact on preventing stunting in children under two years in Angola and improve comparability with other contexts. Robust understanding of the costs and benefits of these types of nutrition strategies is critical for priority setting and to motivate ongoing donor investment and government partnership.

The cost estimation of the different arms of the trial will complement the effectiveness results to provide useful information to the Government of Angola and other policymakers on the most cost-effective and cost-efficient intervention to prevent stunting. Additionally, this study aims to gain understanding on the cost drivers, to promote inclusion of economic evaluations in other nutrition intervention studies, and to improve comparability with other contexts.

One of the limitations of the MuCCUA trial is that it is only powered to test the differences between each of the intervention arms and the comparison group, but not to test the differences between the two intervention arms. The latter would require a larger sample size and resources beyond those available for the trial. However, the possibility of a direct comparison between the interventions will be explored. The protocol has all necessary ethical approvals and the findings of this study will be disseminated within academia and the wider policy sphere.

Author affiliations

[1] Centro Nacional de Medicina Tropical, Instituto de Salud Carlos III, Avda. Monforte de Lemos, 3 Madrid 28029, Spain

[2]Tropical Medicine Unit Vall d'Hebron-Drassanes, Infectious Diseases Department, Vall d'Hebron University Hospital, PROSICS Barcelona, Barcelona 08035, Spain

[3]Centro de Investigación Biomédica en Red de Enfermedades Infecciosas, (CIBERINFEC), Instituto de Salud Carlos III, 28029 Madrid, Spain

[4]Centro Nacional de Epidemiología, Instituto de Salud Carlos III, Avda. Monforte de Lemos, 3 Madrid 28029, Spain

For peer review only - http://bmjopen.bmj.com/site/about/guidelines.xhtml

[5]Fundo Apoio Social-Local Development Institute,Avenida Pedro de Castro Vandunem, Luanda, Angola

[6]Faculdade de Medicina da Universidade Mandume Ya Ndemufayo, Bairro Comercial, Avenida Hoji Ya Haenda nº30, caixa postal 201, Lubango, Huíla, Angola

[7]Action Against Hunger Spain, C/Duque de Sevilla 3, Madrid 28002, Spain

[8]Program in Public Health, Stony Brook University, Stony Brook, New York, USA

Word Count: 4948

Author Contributions

RM-C and EC designed the protocol for the Economic Evaluation and prepared the first draft of the paper. ET, MR-B, ZH, IA, FR, MLF, AS-G, EI, AV, TM, AG, JCL, IM and EC contributed to the conception and design of the MuCCUA trial protocol in which this study is embedded. RM-C, EC and CP designed the cost data collection tools and the processes for allocating costs. ET, IA, FR, MLF, AS-G led the supervision of all intervention implementation activities and contributed to field data acquisition. All authors contributed to the review of this manuscript and provided comments. All authors have read and approve the submitted manuscript.

Acknowledgements

We would like to acknowledge the MuCCUA study participants and field teams including ADECOS, their supervisors and students of the Universidade Mandume ya Ndemufayo for their collaboration in the data collection process. Furthermore, to the Crescer consortium, the FRESAN Program implementers, municipal and province administrations of communes and Huila and Cunene provinces and the health, food and nutrition security and social action offices of the National Government of Angola.

Funding The Crescer project is funded by the UE EuropeAid contract nº FED/2020/418-106 and co-financed by the Crescer consortium partners. The MuCCUA trial is funded by the European Union as a part of the Crescer project (IV component of the FRESAN Program) for the 4-year duration of the project.

Data statement The datasets used and/or analysed during the current study are available from the project coordinator on reasonable request.

For peer review only - http://bmjopen.bmj.com/site/about/guidelines.xhtml

Competing interests None declared.

Ethics approval The research protocol was approved on August 18th, 2022 by the República de Angola Ministério da Saúde Comité de Ética (Instituto Nacional de Investigações em Saúde), reference number 27C.E/MINSA.INIS/2022. The trial was registered in clinical trials on October 7th 2022, NCT05571280.

References

1. Victora CG, Adair L, Fall C, Hallal PC, Martorell R, Richter L, et al. Maternal and child undernutrition: consequences for adult health and human capital. Lancet Lond Engl. 2008 Jan 26;371(9609):340–57.

2. de Onis M, Branca F. Childhood stunting: a global perspective. Matern Child Nutr. 2016;12(S1):12–26.

3. UNICEF/WHO/World Bank Group Joint Child Malnutrition Estimates. Levels and trends in child malnutrition. [Internet]. Available from: https://data.unicef.org/resources/jme-report-2021/

4. Angola Appeal [Internet]. [cited 2022 Jun 6]. Available from: https://www.unicef.org/appeals/angola

5. Angola-Humanitarian-SitRep-June-2022.pdf [Internet]. [cited 2023 Jan 20]. Available from: https://www.unicef.org/media/126971/file/Angola-Humanitarian-SitRep-June-2022.pdf

6. IPC_Angola_FoodSecurity&Nutrition_2021July2022Mar_Snapshot_English.pdf [Internet]. [cited 2022 Mar 28]. Available from: https://www.ipcinfo.org/fileadmin/user_upload/ipcinfo/docs/IPC_Angola_FoodSecurity&Nutrition_2021July2022Mar_Snapshot_English.pdf

7. WFP Angola Country Brief, September 2022 - Angola | ReliefWeb [Internet]. [cited 2023 Jan 20]. Available from: https://reliefweb.int/report/angola/wfp-angola-country-brief-september-2022

8. Governo de Angola, Unicef, World Vision, Technical Rapid Response Team. SMART Nutrition Surveys in drought-affected municipalities of Huila and Cunene provinces in Southern Angola. November-December 2019. 2020.

9. Hess SY, Peerson JM, Becquey E, Abbeddou S, Ouédraogo CT, Somé JW, et al. Differing growth responses to nutritional supplements in neighboring health districts of Burkina Faso are likely due to benefits of small-quantity lipid-based nutrient supplements (LNS). PloS One. 2017;12(8):e0181770.

10. Preventive small-quantity lipid-based nutrient supplements reduce severe wasting and severe stunting among young children: an individual participant data meta-analysis of randomized controlled trials - PubMed [Internet]. [cited 2023 Sep 17]. Available from: https://pubmed.ncbi.nlm.nih.gov/36045000/

For peer review only - http://bmjopen.bmj.com/site/about/guidelines.xhtml

11. Carneiro P, Kraftman L, Mason G, Moore L, Rasul I, Scott M. The Impacts of a Multifaceted Prenatal Intervention on Human Capital Accumulation in Early Life. Am Econ Rev. 2021 Aug 1;111(8):2506–49.

12. Cooper JE, Benmarhnia T, Koski A, King NB. Cash transfer programs have differential effects on health: A review of the literature from low and middle-income countries. Soc Sci Med 1982. 2020 Jan 25;247:112806.

13. Ministério da Saude, República de Angola. Política Nacional de Agentes de Desenvolvimento Comunitário E Sanitário (ADECOS). Luanda, Angola; 2014.

14. Hospital Universitari Vall d'Hebron Research Institute. Maternal and Child Interventions Against Chronic Malnutrition in Angola. A Cluster-randomized Cost-benefit Community-based Study. [Internet]. clinicaltrials.gov; 2023 Jan [cited 2023 Feb 2]. Report No.: NCT05571280. Available from: https://clinicaltrials.gov/ct2/show/NCT05571280

15. Heidkamp RA, Piwoz E, Gillespie S, Keats EC, D'Alimonte MR, Menon P, et al. Mobilising evidence, data, and resources to achieve global maternal and child undernutrition targets and the Sustainable Development Goals: an agenda for action. The Lancet. 2021 Apr 10;397(10282):1400–18.

16. Adu-Afarwuah S, Lartey A, Brown KH, Zlotkin S, Briend A, Dewey KG. Randomized comparison of 3 types of micronutrient supplements for home fortification of complementary foods in Ghana: effects on growth and motor development. Am J Clin Nutr. 2007 Aug 1;86(2):412–20.

17. Phuka JC, Maleta K, Thakwalakwa C, Cheung YB, Briend A, Manary MJ, et al. Postintervention growth of Malawian children who received 12-mo dietary complementation with a lipid-based nutrient supplement or maize-soy flour. Am J Clin Nutr. 2009 Jan 1;89(1):382–90.

18. Hambidge KM, Westcott JE, Garcés A, Figueroa L, Goudar SS, Dhaded SM, et al. A multicountry randomized controlled trial of comprehensive maternal nutrition supplementation initiated before conception: the Women First trial. Am J Clin Nutr. 2019 Feb 1;109(2):457–69.

19. Levere M, Acharya G, Bharadwaj P. The Role of Information and Cash Transfers on Early Childhood Development: Evidence from Nepal. Cambridge, Mass: National Bureau of Economic Research; 2016. 1 p. (NBER working paper series).

20. Fenn B, Colbourn T, Dolan C, Pietzsch S, Sangrasi M, Shoham J. Impact evaluation of different cash-based intervention modalities on child and maternal nutritional status in Sindh Province, Pakistan, at 6 mo and at 1 y: A cluster randomised controlled trial. PLoS Med. 2017 May;14(5):e1002305.

21. Keats EC, Das JK, Salam RA, Lassi ZS, Imdad A, Black RE, et al. Effective interventions to address maternal and child malnutrition: an update of the evidence. Lancet Child Adolesc Health. 2021 May 1;5(5):367–84.

22. The Impacts of a Multifaceted Prenatal Intervention on Human Capital Accumulation in Early Life - American Economic Association [Internet]. [cited 2023 Sep 17]. Available from: https://www.aeaweb.org/articles?id=10.1257/aer.20191726

For peer review only - http://bmjopen.bmj.com/site/about/guidelines.xhtml

23. Ogada, Edna, Bahwere, Paluku, Lelijveld, Natasha, Sessions, Natalie, Desplats, Gwenola, Khara, Tanya. Multi-sectoral Nutrition Programming – Exploring Impact. ENN Publication [Internet]. ENN_7135. Available from: www.ennonline.net/mspexploringimpact

24. Ruel MT, Alderman H. Nutrition-sensitive interventions and programmes: how can they help to accelerate progress in improving maternal and child nutrition? The Lancet. 2013 Aug;382(9891):536–51.

25. Wun J, Kemp C, Puett C, Bushnell D, Crocker J, Levin C. Measurement of benefits in economic evaluations of nutrition interventions in low- and middle-income countries: A systematic review. Matern Child Nutr. 2022;18(2):e13323.

26. Batura N, Hill Z, Haghparast-Bidgoli H, Lingam R, Colbourn T, Kim S, et al. Highlighting the evidence gap: how cost-effective are interventions to improve early childhood nutrition and development? Health Policy Plan. 2015 Jul 1;30(6):813–21.

27. Remme M, Martinez-Alvarez M, Vassall A. Cost-Effectiveness Thresholds in Global Health: Taking a Multisectoral Perspective. Value Health. 2017 Apr 1;20(4):699–704.

28. Ramponi F, Tafesse W, Griffin S. Economic evaluation of interventions to address undernutrition: a systematic review. Health Policy Plan. 2021 May 1;36(4):533–41.

29. Drummond M, Sculpher MJ, Claxton K, Stoddart GL, Torrance GW. Methods for the Economic Evaluation of Health Care Programmes. Oxford University Press; 2015. 461 p.

30. Drummond M, Stoddart G. Assessment of health producing measures across different sectors. Health Policy Amst Neth. 1995 Sep;33(3):219–31.

31. Jönsson B. Ten arguments for a societal perspective in the economic evaluation of medical innovations. Eur J Health Econ. 2009 Oct 1;10(4):357–9.

32. Byford S, Raftery J. Perspectives in economic evaluation. BMJ. 1998 May 16;316(7143):1529–30.

33. Richter, Susan, Iruhiriye, Elyse, Heckert, Jessica, Sununtnasuk, Celeste, Ruel, Marie, Leroy, Jef, et al. Cost study of the Preventing Malnutrition in Children Under 2 Years of Age Approach in Burundi and Guatemala. Washington DC;

34. Heckert J, Leroy JL, Olney DK, Richter S, Iruhiriye E, Ruel MT. The cost of improving nutritional outcomes through food-assisted maternal and child health and nutrition programmes in Burundi and Guatemala. Matern Child Nutr. 2020 Jan;16(1):e12863.

35. Trenouth L, Colbourn T, Fenn B, Pietzsch S, Myatt M, Puett C. The cost of preventing undernutrition: cost, cost-efficiency and cost-effectiveness of three cash-based interventions on nutrition outcomes in Dadu, Pakistan. Health Policy Plan. 2018 Jul 1;33(6):743–54.

36. Njuguna RG, Berkley JA, Jemutai J. Cost and cost-effectiveness analysis of treatment for child undernutrition in low- and middle-income countries: A systematic review. Wellcome Open Res. 2020 Oct 5;5:62.

37. SEEMS-Nutrition [Internet]. [cited 2023 Jan 31]. Available from: https://sites.google.com/uw.edu/seems-nutrition/home

For peer review only - http://bmjopen.bmj.com/site/about/guidelines.xhtml

38. Instituto Nacional de Estatística [Internet]. [cited 2022 Jun 6]. Available from: https://www.ine.gov.ao/

39. Instituto Nacional de Estadística, Luanda-Angola. POBREZA MULTIDIMENSIONAL NOS MUNICÍPIOS DE ANGOLA [Internet]. 2019. Available from: https://www.ine.gov.ao/Arquivos/arquivosCarregados//Carregados/Publicacao_637586747742336023.pdf

40. Plano de Desenvolvimento Nacional 2018-2022. Governo de Angola [Internet]. [cited 2023 Sep 26]. Available from: https://www.ucm.minfin.gov.ao/cs/groups/public/documents/document/zmlu/njax/~edisp/minfin601408.pdf

41. Ministério da Saude, República de Angola. Plano estratégico multissectorial de nutrição 2019-2025. Luanda, Angola; 2019.

42. Freedland KE, King AC, Ambrosius WT, Mayo-Wilson E, Mohr DC, Czajkowski SM, et al. The selection of comparators for randomized controlled trials of health-related behavioral interventions: recommendations of an NIH expert panel. J Clin Epidemiol. 2019 Jun;110:74–81.

43. Kaplan RS, Anderson SR. Time-driven activity-based costing. Harv Bus Rev. 2004 Nov;82(11):131–8, 150.

44. Johns B, Baltussen R, Hutubessy R. Programme costs in the economic evaluation of health interventions. Cost Eff Resour Alloc CE. 2003 Feb 26;1(1):1.

45. Batura N, Pulkki-Brännström AM, Agrawal P, Bagra A, Haghparast- Bidgoli H, Bozzani F, et al. Collecting and analysing cost data for complex public health trials: reflections on practice. Glob Health Action. 2014 Dec 1;7(1):23257.

46. Hutton G, Baltussen R. Cost valuation in resource-poor settings. Health Policy Plan. 2005 Jul 1;20(4):252–9.

47. Walker D. Cost and cost-effectiveness guidelines: which ones to use? Health Policy Plan. 2001 Mar 1;16(1):113–21.

48. World Health Organization, Baltussen RMPM, Adam T, Tan-Torres Edejer T, Hutubessy RCW, Acharya A, et al. Making choices in health : WHO guide to cost-effectiveness analysis [Internet]. World Health Organization; 2003 [cited 2022 Jun 13]. Available from: https://apps.who.int/iris/handle/10665/42699

49. Claxton KP, Revill P, Sculpher M, Wilkinson T, Cairns J, Briggs A. The Gates Reference Case for Economic Evaluation. [Internet]. The Bill and Melinda Gates Foundation; 2014. Available from: https://www.idsihealth.org/wp-content/uploads/2016/05/Gates-Reference-case-what-it-is-how-to-use-it.pdf

For peer review only

For peer review only - http://bmjopen.bmj.com/site/about/guidelines.xhtml

Reporting checklist for protocol of a clinical trial.

Based on the SPIRIT guidelines.

Instructions to authors

Complete this checklist by entering the page numbers from your manuscript where readers will find each of the items listed below.

Your article may not currently address all the items on the checklist. Please modify your text to include the missing information. If you are certain that an item does not apply, please write "n/a" and provide a short explanation.

Upload your completed checklist as an extra file when you submit to a journal.

In your methods section, say that you used the SPIRITreporting guidelines, and cite them as:

Chan A-W, Tetzlaff JM, Gøtzsche PC, Altman DG, Mann H, Berlin J, Dickersin K, Hróbjartsson A, Schulz KF, Parulekar WR, Krleža-Jerić K, Laupacis A, Moher D. SPIRIT 2013 Explanation and Elaboration: Guidance for protocols of clinical trials. BMJ. 2013;346:e7586

		Reporting Item	Page Number
**Administrative information**			
Title	#1	Descriptive title identifying the study design, population, interventions, and, if applicable, trial acronym	1

For peer review only

Trial registration	#2a	Trial identifier and registry name. If not yet registered, name of intended registry	2
Trial registration: data set	#2b	All items from the World Health Organization Trial Registration Data Set	All items addressed
Protocol version	#3	Date and version identifier	
Funding	#4	Sources and types of financial, material, and other support	21
Roles and responsibilities: contributorship	#5a	Names, affiliations, and roles of protocol contributors	1, 20-21
Roles and responsibilities: sponsor contact information	#5b	Name and contact information for the trial sponsor	1
Roles and responsibilities: sponsor and funder	#5c	Role of study sponsor and funders, if any, in study design; collection, management, analysis, and interpretation of data; writing of the report; and the decision to submit the report for publication, including whether they will have ultimate authority over any of these activities	

For peer review only

Roles and responsibilities: committees	#5d	Composition, roles, and responsibilities of the coordinating centre, steering committee, endpoint adjudication committee, data management team, and other individuals or groups overseeing the trial, if applicable (see Item 21a for data monitoring committee)	N/A
**Introduction**			
Background and rationale	#6a	Description of research question and justification for undertaking the trial, including summary of relevant studies (published and unpublished) examining benefits and harms for each intervention	3
Background and rationale: choice of comparators	#6b	Explanation for choice of comparators	5
Objectives	#7	Specific objectives or hypotheses	6
Trial design	#8	Description of trial design including type of trial (eg, parallel group, crossover, factorial, single group), allocation ratio, and framework (eg, superiority, equivalence, non-inferiority, exploratory)	4,7, 9

For peer review only

Methods: Participants, interventions, and outcomes

Study setting	#9	Description of study settings (eg, community clinic, academic hospital) and list of countries where data will be collected. Reference to where list of study sites can be obtained	8
Eligibility criteria	#10	Inclusion and exclusion criteria for participants. If applicable, eligibility criteria for study centres and individuals who will perform the interventions (eg, surgeons, psychotherapists)	9
Interventions: description	#11a	Interventions for each group with sufficient detail to allow replication, including how and when they will be administered	9-11
Interventions: modifications	#11b	Criteria for discontinuing or modifying allocated interventions for a given trial participant (eg, drug dose change in response to harms, participant request, or improving / worsening disease)	N/A to economic evaluation
Interventions: adherance	#11c	Strategies to improve adherence to intervention protocols, and any	N/A to economic evaluation

For peer review only

		procedures for monitoring adherence (eg, drug tablet return; laboratory tests)	
Interventions: concomitant care	#11d	Relevant concomitant care and interventions that are permitted or prohibited during the trial	N/A to economic evaluation
Outcomes	#12	Primary, secondary, and other outcomes, including the specific measurement variable (eg, systolic blood pressure), analysis metric (eg, change from baseline, final value, time to event), method of aggregation (eg, median, proportion), and time point for each outcome. Explanation of the clinical relevance of chosen efficacy and harm outcomes is strongly recommended	11-12
Participant timeline	#13	Time schedule of enrolment, interventions (including any run-ins and washouts), assessments, and visits for participants. A schematic diagram is highly recommended (see Figure)	9
Sample size	#14	Estimated number of participants needed to achieve study objectives and how it was determined, including clinical and statistical assumptions supporting any sample size calculations	9

For peer review only

Recruitment	#15	Strategies for achieving adequate participant enrolment to reach target sample size	N/A
**Methods: Assignment of interventions (for controlled trials)**			
Allocation: sequence generation	#16a	Method of generating the allocation sequence (eg, computer-generated random numbers), and list of any factors for stratification. To reduce predictability of a random sequence, details of any planned restriction (eg, blocking) should be provided in a separate document that is unavailable to those who enrol participants or assign interventions	Randomisation of main trial is mentioned briefly on pg 8 and main trial protocol referenced for more detail; sampling for Economic evaluation components is explained in Table 1
Allocation concealment mechanism	#16b	Mechanism of implementing the allocation sequence (eg, central telephone; sequentially numbered, opaque, sealed envelopes), describing any steps to conceal the sequence until interventions are assigned	Main trial protocol is referenced for more detail.
Allocation: implementation	#16c	Who will generate the allocation sequence, who will enrol participants, and	Main trial protocol is referenced for more detail.

For peer review only

		who will assign participants to interventions	
Blinding (masking)	#17a	Who will be blinded after assignment to interventions (eg, trial participants, care providers, outcome assessors, data analysts), and how	Main trial protocol is referenced for more detail.
Blinding (masking): emergency unblinding	#17b	If blinded, circumstances under which unblinding is permissible, and procedure for revealing a participant's allocated intervention during the trial	N/A
**Methods: Data collection, management, and analysis**			
Data collection plan	#18a	Plans for assessment and collection of outcome, baseline, and other trial data, including any related processes to promote data quality (eg, duplicate measurements, training of assessors) and a description of study instruments (eg, questionnaires, laboratory tests) along with their reliability and validity, if known. Reference to where data collection forms can be found, if not in the protocol	11-13

For peer review only

Data collection plan: retention	#18b	Plans to promote participant retention and complete follow-up, including list of any outcome data to be collected for participants who discontinue or deviate from intervention protocols	N/A
Data management	#19	Plans for data entry, coding, security, and storage, including any related processes to promote data quality (eg, double data entry; range checks for data values). Reference to where details of data management procedures can be found, if not in the protocol	Detail provided in the main trial protocol.
Statistics: outcomes	#20a	Statistical methods for analysing primary and secondary outcomes. Reference to where other details of the statistical analysis plan can be found, if not in the protocol	18-19
Statistics: additional analyses	#20b	Methods for any additional analyses (eg, subgroup and adjusted analyses)	12
Statistics: analysis population and missing data	#20c	Definition of analysis population relating to protocol non-adherence (eg, as randomised analysis), and any statistical methods to handle missing data (eg, multiple imputation)	N/A

For peer review only

Methods: Monitoring

Data monitoring: formal committee	#21a	Composition of data monitoring committee (DMC); summary of its role and reporting structure; statement of whether it is independent from the sponsor and competing interests; and reference to where further details about its charter can be found, if not in the protocol. Alternatively, an explanation of why a DMC is not needed	N/A to economic evaluation
Data monitoring: interim analysis	#21b	Description of any interim analyses and stopping guidelines, including who will have access to these interim results and make the final decision to terminate the trial	N/A
Harms	#22	Plans for collecting, assessing, reporting, and managing solicited and spontaneously reported adverse events and other unintended effects of trial interventions or trial conduct	N/A to economic evaluation
Auditing	#23	Frequency and procedures for auditing trial conduct, if any, and whether the	N/A to economic evaluation

For peer review only

		process will be independent from investigators and the sponsor	
**Ethics and dissemination**			
Research ethics approval	#24	Plans for seeking research ethics committee / institutional review board (REC / IRB) approval	21
Protocol amendments	#25	Plans for communicating important protocol modifications (eg, changes to eligibility criteria, outcomes, analyses) to relevant parties (eg, investigators, REC / IRBs, trial participants, trial registries, journals, regulators)	N/A to economic evaluation
Consent or assent	#26a	Who will obtain informed consent or assent from potential trial participants or authorised surrogates, and how (see Item 32)	8
Consent or assent: ancillary studies	#26b	Additional consent provisions for collection and use of participant data and biological specimens in ancillary studies, if applicable	N/A
Confidentiality	#27	How personal information about potential and enrolled participants will be collected, shared, and maintained in order to protect	21

For peer review only

		confidentiality before, during, and after the trial	
Declaration of interests	#28	Financial and other competing interests for principal investigators for the overall trial and each study site	21
Data access	#29	Statement of who will have access to the final trial dataset, and disclosure of contractual agreements that limit such access for investigators	21
Ancillary and post trial care	#30	Provisions, if any, for ancillary and post-trial care, and for compensation to those who suffer harm from trial participation	N/A
Dissemination policy: trial results	#31a	Plans for investigators and sponsor to communicate trial results to participants, healthcare professionals, the public, and other relevant groups (eg, via publication, reporting in results databases, or other data sharing arrangements), including any publication restrictions	7
Dissemination policy: authorship	#31b	Authorship eligibility guidelines and any intended use of professional writers	N/A
Dissemination policy:	#31c	Plans, if any, for granting public access to the full protocol, participant-level dataset, and statistical code	N/A

For peer review only

reproducible research			

Appendices

Informed consent materials	#32	Model consent form and other related documentation given to participants and authorised surrogates	-
Biological specimens	#33	Plans for collection, laboratory evaluation, and storage of biological specimens for genetic or molecular analysis in the current trial and for future use in ancillary studies, if applicable	N/A

None The SPIRIT Explanation and Elaboration paper is distributed under the terms of the Creative Commons Attribution License CC-BY-NC. This checklist can be completed online using https://www.goodreports.org/, a tool made by the EQUATOR Network in collaboration with Penelope.ai

For peer review only

CHEERS 2022 Checklist

**Title**			
Title	1	Identify the study as an economic evaluation and specify the interventions being compared.	1
**Abstract**			
Abstract	2	Provide a structured summary that highlights context, key methods, results, and alternative analyses.	1-2
**Introduction**			
Background and objectives	3	Give the context for the study, the study question, and its practical relevance for decision making in policy or practice.	3-7
Methods			
**Health economic analysis plan**	**4**	**Indicate whether a health economic analysis plan was developed and where available.**	**7**
Study population	5	Describe characteristics of the study population (such as age range, demographics, socioeconomic, or clinical characteristics).	8
Setting and location	6	Provide relevant contextual information that may influence findings.	8
Comparators	7	Describe the interventions or strategies being compared and why chosen.	9-11
Perspective	8	State the perspective(s) adopted by the study and why chosen.	12
Time horizon	9	State the time horizon for the study and why appropriate.	9

For peer review only - http://bmjopen.bmj.com/site/about/guidelines.xhtml
For peer review only

(continued)

Discount rate	10	Report the discount rate(s) and reason chosen.	19
Selection of outcomes	11	Describe what outcomes were used as the measure(s) of benefit(s) and harm(s).	11-12
Measurement of outcomes	12	Describe how outcomes used to capture benefit(s) and harm(s) were measured.	11-12
Valuation of outcomes	13	Describe the population and methods used to measure and value outcomes.	11-12
Measurement and valuation of resources and costs	14	Describe how costs were valued.	12-18
Currency, price date, and conversion	15	Report the dates of the estimated resource quantities and unit costs, plus the currency and year of conversion.	19
Rationale and description of model	16	If modelling is used, describe in detail and why used. Report if the model is publicly available and where it can be accessed.	N/A
Analytics and assumptions	17	Describe any methods for analysing or statistically transforming data, any extrapolation methods, and approaches for validating any model used.	12-18
Characterising heterogeneity	18	Describe any methods used for estimating how the results of the study vary for subgroups.	12
Characterising distributional effects	19	Describe how impacts are distributed across different individuals or adjustments made to reflect priority populations.	12

For peer review only

(continued)

Characterising uncertainty	20	Describe methods to characterise any sources of uncertainty in the analysis.	19
Approach to engagement with patients and others affected by the study	21	Describe any approaches to engage patients or service recipients, the general public, communities, or stakeholders (such as clinicians or payers) in the design of the study.	7
**Results**			
Study parameters	22	Report all analytic inputs (such as values, ranges, references) including uncertainty or distributional assumptions.	18-19
Summary of main results	23	Report the mean values for the main categories of costs and outcomes of interest and summarise them in the most appropriate overall measure.	18-19
Effect of uncertainty	24	Describe how uncertainty about analytic judgments, inputs, or projections affect findings. Report the effect of choice of discount rate and time horizon, if applicable.	12
Effect of engagement with patients and others affected by the study	25	Report on any difference patient/service recipient, general public, community, or stakeholder involvement made to the approach or findings of the study	12
Discussion			

For peer review only - http://bmjopen.bmj.com/site/about/guidelines.xhtml

(continued)

Study findings, limitations, generalisability, and current knowledge	26	Report key findings, limitations, ethical or equity considerations not captured, and how these could affect patients, policy, or practice.	19-20
Other relevant information			
Source of funding	27	Describe how the study was funded and any role of the funder in the identification, design, conduct, and reporting of the analysis	19-20
Conflicts of interest	28	Report authors conflicts of interest according to journal or International Committee of Medical Journal Editors requirements.	21

From: Husereau D, Drummond M, Augustovski F, et al. Consolidated Health Economic Evaluation Reporting Standards 2022 (CHEERS 2022) Explanation and Elaboration: A Report of the ISPOR CHEERS II Good Practices Task Force. Value Health 2022;25. doi:10.1016/j.jval.2021.10.008

For peer review only
